# Supplementary material for: Variability in the use of pulse oximeters with children in Kenyan hospitals: A mixed-methods analysis
Source: PLoS Med. 2019 Dec 31;16(12):e1002987. doi: 10.1371/journal.pmed.1002987 (PMC6938307; doi:10.1371/journal.pmed.1002987)
Supplement: S1 Table — CIN, Clinical Information Network. (DOCX) [file pmed.1002987.s001.docx]

S1 Table. Characteristics of the CIN hospitals from which quantitative and qualitative data were collected

| Characteristics | Logistic regressions | Direct observations | Longer audio- recorded semi- structured interviews | Shorter interviews with HCWs at CIN meeting | Shorter interviews with Medical Superintendent and Procurement Officials |
| --- | --- | --- | --- | --- | --- |
| Number of hospitals | 7 | 3 | 4 | 10 | 2 |
| IDs of hospitals | #1-7 | #5, #6, #12 | #5, #7, #11, #12 | #1, #2, #3, #4, #6, #8, #9, #10, #13, #14 | #5, #12 |
| Number of counties | 6 | 2 | 4 | 10 | 2 |
| Paediatric ward capacities (# beds) | 29-63 | 35-42 | 30-60 | 21-63 | 35-42 |
| % of catchment area populations living in extreme poverty | 20-50% | 20-55% | 20-55% | 20-65% | 20-55% |
| % of children obtaining pulse oximeter reading on average between Sept 2013 and Feb 2016 | 21-75% | 0-75% | 0-75% | 0-52% | 0-75% |

[1]

References:

1. Ayieko P, Ogero M, Makone B, Julius T, Mbevi G, Nyachiro W, et al. Characteristics of admissions and variations in the use of basic investigations, treatments and outcomes in Kenyan hospitals within a new Clinical Information Network. Archives of Disease in Childhood 2016;101:223-229.
