# Supplementary material for: Variability in the use of pulse oximeters with children in Kenyan hospitals: A mixed-methods analysis
Source: PLoS Med. 2019 Dec 31;16(12):e1002987. doi: 10.1371/journal.pmed.1002987 (PMC6938307; doi:10.1371/journal.pmed.1002987)
Supplement: S2 Table — CIN, Clinical Information Network. (DOCX) [file pmed.1002987.s002.docx]

S2 Table. Descriptive statistics of the children admitted to the seven CIN hospitals during the study period

| **Variable** | **Value** | **Proportion with each variable value** | **% with whom a pulse oximeter was used** | **Missing data level** |
| --- | --- | --- | --- | --- |
| Pulse oximeter use | A pulse oximeter was used | 51% | 100% | <5% |
|  | A pulse oximeter was not used | 49% | 0% |  |
| Pulse oximeter value | Had an oxygen saturation value <90% | 11% | 100% | 5% ≤ x < 10% |
|  | Had an oxygen saturation value ≥90% | 89% | 100% |  |
| Oxygen use | Were prescribed oxygen | 8% | 58 | <5% |
|  | Were not prescribed oxygen | 92% | 50 |  |
| Month of admission | January | 9% of total admissions | 55 | <5% |
|  | February | 12% | 50 |  |
|  | March | 10% | 53 |  |
|  | April | 8% | 35 |  |
|  | May | 9% | 33 |  |
|  | June | 9% | 45 |  |
|  | July | 7% | 54 |  |
|  | August | 7% | 50 |  |
|  | September | 6% | 53 |  |
|  | October | 8% | 58 |  |
|  | November | 9% | 66 |  |
|  | December | 4% | 69 |  |
| Weekend admission | Admitted on a weekend | 25% | 50 | <5% |
|  | Admitted on a weekday | 75% | 51 |  |
| Admission time period | Sept 2013-Feb 2014 | 12% of total admissions | 43 | <5% |
|  | March 2014-Aug 2014 | 29% | 31 |  |
|  | Sept 2014-Feb 2015 | 19% | 54 |  |
|  | March 2015-Aug 2015 | 22% | 59 |  |
|  | Sept 2015-Feb 2016 | 18% | 70 |  |
| Hospital | 1 | 4,299 admissions (15% of total) | 52 | <5% |
|  | 2 | 2,727 (10%) | 21 |  |
|  | 3 | 3,957 (14%) | 47 |  |
|  | 4 | 5,734 (21%) | 42 |  |
|  | 5 | 3,845 (14%) | 75 |  |
|  | 6 | 3,992 (14%) | 44 |  |
|  | 7 | 3,360 (12%) | 74 |  |
| Hospital use of pulse oximeters by admission month | A month that pulse oximeters were used with 0-20% of children at that particular hospital | 23% of admissions | 0-20% |  |
|  | 21-50% pulse oximeter use | 21% | 21-50% |  |
|  | 51-80% pulse oximeter use | 37% | 51-80% |  |
|  | 81-100% pulse oximeter use | 19% | 81-100% |  |
| Sex | Female | 45% | 51% | <5% |
|  | Male | 55% | 51% |  |
| Age in years | n/a | Mean: 2.26  Median: 1  Range 0 - 12 | n/a | <5% |
| Weight-for-age | n/a | Mean: -0.83  Median: -0.63  Range: -10.33 - 22.04 | n/a | 5% ≤ x < 10% |
| Paediatric Admission Record (PAR) use | No PAR available | 5% | 37% | 20% ≤ x ≤ 25% |
|  | PAR present, not used | 6% | 53% |  |
|  | PAR used | 89% | 59% |  |
| Length of illness before admission | 1 day | 25% | 53% | 5% ≤ x < 10% |
|  | 2 days | 18% | 52% |  |
|  | 3 days | 20% | 51% |  |
|  | 4 days | 9% | 50% |  |
|  | 4+ days | 27% | 51% |  |
| Fever | Yes | 75% | 52% | 5% ≤ x < 10% |
|  | No | 25% | 52% |  |
| Cough | Yes | 58% | 53% | 5% ≤ x < 10% |
|  | No | 42% | 52% |  |
| Difficulty breathing | Yes | 34% | 54% | 10% ≤ x < 15% |
|  | No | 66% | 53% |  |
| Vomiting everything | Yes | 21% | 48% | 10% ≤ x < 15% |
|  | No | 79% | 55% |  |
| Difficulty feeding | Yes | 34% | 51% | 10% ≤ x < 15% |
|  | No | 66% | 55% |  |
| Convulsions | Yes | 20% | 52% | 10% ≤ x < 15% |
|  | No | 80% | 53% |  |
| Very high respiratory rate | Yes | 11% | 60% | 20% ≤ x ≤ 25% |
|  | No | 89% | 57% |  |
| Oedema | Yes | 3% | 47% | 10% ≤ x < 15% |
|  | No | 97% | 53% |  |
| Stridor | Yes | 3% | 57% | 10% ≤ x < 15% |
|  | No | 97% | 54% |  |
| Central cyanosis | Yes | 1% | 58% | 5% ≤ x < 10% |
|  | No | 99% | 53% |  |
| Chest indrawing | Yes | 32% | 54% | 10% ≤ x < 15% |
|  | No | 68% | 53% |  |
| Grunting | Yes | 12% | 54% | 10% ≤ x < 15% |
|  | No | 88% | 54% |  |
| Wheeze | Yes | 7% | 52% | 10% ≤ x < 15% |
|  | No | 93% | 53% |  |
| Crackles | Yes | 22% | 54% | 10% ≤ x < 15% |
|  | No | 78% | 52% |  |
| Capillary refill | Had a capillary refill >3 seconds | 1% | 56% | 20% ≤ x ≤ 25% |
|  | Capillary refill ≤3 seconds | 99% | 55% |  |
| Pallor | Yes | 14% | 50% | 5% ≤ x < 10% |
|  | No | 86% | 53% |  |
| Lack of alertness | Not alert | 7% | 53% | 5% ≤ x < 10% |
|  | Alert | 93% | 52% |  |
| Difficulty drinking | Yes | 17% | 53% | 15% ≤ x < 20% |
|  | No | 83% | 54% |  |
| Stiff neck | Yes | 3% | 51% | 10% ≤ x < 15% |
|  | No | 97% | 53% |  |
| Bulging fontanelle | Yes | 1% | 47% | 10% ≤ x < 15% |
|  | No | 99% | 54% |  |
| Diagnosis pneumonia | Yes | 45% | 54% | <5% |
|  | No | 55% | 48% |  |
| Diagnosis malaria | Yes | 28% | 45% | <5% |
|  | No | 72% | 53% |  |
| Diagnosis TB | Yes | 2% | 54% | <5% |
|  | No | 98% | 51% |  |
| Diagnosis diarrhoea | Yes | 35% | 49% | <5% |
|  | No | 65% | 52% |  |
| Diagnosis dehydration | Yes | 20% | 49% | <5% |
|  | No | 80% | 51% |  |
| Diagnosis malnutrition | Yes | 10% | 50% | <5% |
|  | No | 90% | 51% |  |
| Diagnosis anaemia | Yes | 8% | 50% | <5% |
|  | No | 92% | 51% |  |
| Diagnosis meningitis | Yes | 10% | 51% | <5% |
|  | No | 90% | 51% |  |
| Diagnosis asthma | Yes | 3% | 61% | <5% |
|  | No | 97% | 50% |  |
| Diagnosis bronchiolitis | Yes | 2% | 48% | <5% |
|  | No | 98% | 51% |  |
| Diagnosis sepsis | Yes | 2% | 52% | <5% |
|  | No | 98% | 51% |  |
| Antimalarials | Were given oral antimalarials | 2% | 43% | <5% |
|  | Injectable antimalarials | 22% | 44% |  |
|  | Neither | 75% | 53% |  |
| Outcome | Alive | 91% | 51% | <5% |
|  | Died | 6% | 51% |  |
|  | Referred | 2% | 63% |  |
|  | Other | 1% | 42% |  |
