# Supplementary material for: Variability in the use of pulse oximeters with children in Kenyan hospitals: A mixed-methods analysis
Source: PLoS Med. 2019 Dec 31;16(12):e1002987. doi: 10.1371/journal.pmed.1002987 (PMC6938307; doi:10.1371/journal.pmed.1002987)
Supplement: S3 Table — OR, odds ratio. (DOCX) [file pmed.1002987.s003.docx]

S3 Table. Odds ratios and confidence intervals produced from the logistic regression investigating with which children pulse oximeters are used

| **Variable** | **Baseline** | **Odds ratio** | **Confidence interval** |
| --- | --- | --- | --- |
| Intercept | n/a | 0.02 | 0.01, 0.02 |
| Hospital 2 | Hospital 1 | 0.82 | 0.70, 0.95 |
| Hospital 3 |  | 1.39 | 1.22, 1.57 |
| Hospital 4 |  | 0.97 | 0.88, 1.07 |
| Hospital 5 |  | 1.16 | 1.04, 1.30 |
| Hospital 6 |  | 0.95 | 0.85, 1.06 |
| Hospital 7 |  | 1.29 | 1.13, 1.47 |
| Female | Male | 0.99 | 0.93, 1.05 |
| Age | n/a | 0.98 | 0.97, 0.99 |
| Weight-for-age | n/a | 1.01 | 0.99, 1.02 |
| March 2014-Aug 2014 | Sept 2013 –  Feb 2014 | 1.22 | 0.98, 1.52 |
| Sept 2014-Feb 2015 |  | 1.39 | 1.09, 1.78 |
| March 2015-Aug 2015 |  | 1.49 | 1.18, 1.88 |
| Sept 2015-Feb 2016 |  | 1.59 | 1.26, 2.02 |
| 21-50% pulse oximeter use^i^ | 0-20% pulse oximeter use | 10.49 | 9.09, 12.09 |
| 51-80% pulse oximeter use^i^ |  | 30.38 | 26.16, 35.28 |
| 81-100% pulse oximeter use^i^ |  | 101.40 | 85.79, 119.85 |
| PAR present not used | PAR not present | 2.93 | 2.05, 4.19 |
| PAR used |  | 2.41 | 1.98, 2.94 |
| Weekend admission | Weekday admission | 0.91 | 0.85, 0.98 |
| Fever | No fever | 1.12 | 1.04, 1.20 |
| Cough | No cough | 1.12 | 1.04, 1.21 |
| Difficulty breathing | No difficulty breathing | 1.13 | 1.05, 1.22 |
| Vomit everything | Not vomiting everything | 0.84 | 0.78, 0.91 |
| Very high respiratory rate | Low/normal respiratory rate | 1.27 | 1.13, 1.43 |
| Chest indrawing | No indrawing | 1.28 | 1.17, 1.40 |
| Difficulty drinking | No difficulty drinking | 0.89 | 0.80, 0.98 |
| Not alert | Alert | 1.30 | 1.09, 1.55 |
| Indrawing x Not alert | n/a | 0.75 | 0.57, 0.98 |

^i^ The level of pulse oximeter use at the hospital during the month of admission of the child
