# Supplementary material for: Variability in the use of pulse oximeters with children in Kenyan hospitals: A mixed-methods analysis
Source: PLoS Med. 2019 Dec 31;16(12):e1002987. doi: 10.1371/journal.pmed.1002987 (PMC6938307; doi:10.1371/journal.pmed.1002987)
Supplement: S4 Table — OR, odds ratio. (DOCX) [file pmed.1002987.s004.docx]

S4 Table. Odds ratios and confidence intervals produced from the logistic regression investigating the factors influencing whether oxygen is prescribed

| **Variable** | **Baseline** | **Odds ratio** | **Confidence interval** |
| --- | --- | --- | --- |
| Intercept | n/a | 0.04 | 0.02, 0.07 |
| Pulse oximeter use | No pulse oximeter used | 1.42 | 1.25, 1.62 |
| Hospital 2 | Hospital 1 | 0.36 | 0.29, 0.45 |
| Hospital 3 |  | 0.47 | 0.36, 0.61 |
| Hospital 4 |  | 0.87 | 0.72, 1.06 |
| Hospital 5 |  | 0.91 | 0.76, 1.09 |
| Hospital 6 |  | 0.69 | 0.59, 0.82 |
| Hospital 7 |  | 0.55 | 0.45, 0.69 |
| Female | Male | 1.09 | 0.98, 1.20 |
| Age years | n/a | 0.95 | 0.92, 0.97 |
| Weight-for-age | n/a | 0.98 | 0.95, 1.01 |
| March 2014-Aug 2014 | Sept 2013 – Feb 2014 | 0.46 | 0.32, 0.65 |
| Sept 2014-Feb 2015 |  | 0.51 | 0.35, 0.74 |
| March 2015-Aug 2015 |  | 0.55 | 0.38, 0.79 |
| Sept 2015-Feb 2016 |  | 0.60 | 0.41, 0.87 |
| 21-50% pulse oximeter use | 0-20% pulse oximeter use | 0.71 | 0.60, 0.85 |
| 51-80% pulse oximeter use |  | 0.53 | 0.45, 0.64 |
| 81-100% pulse oximeter use |  | 0.55 | 0.44, 0.69 |
| PAR present not used | PAR not present | 1.08 | 0.65, 1.82 |
| PAR used |  | 1.36 | 0.91, 2.03 |
| Weekend admission | Weekday admission | 1.09 | 0.98, 1.22 |
| Fever | No fever | 0.79 | 0.70, 0.89 |
| Cough | No cough | 1.09 | 0.95, 1.26 |
| Difficulty breathing | No difficulty breathing | 2.00 | 1.76, 2.28 |
| Vomit everything | Not vomiting everything | 0.87 | 0.75, 1.01 |
| Difficulty feeding | No difficulty feeding | 0.93 | 0.83, 1.04 |
| Convulsions | No convulsions | 0.85 | 0.70, 1.04 |
| Very high respiratory rate | Low/normal respiratory rate | 1.87 | 1.64, 2.14 |
| Cyanosis | No cyanosis | 1.92 | 1.34, 2.76 |
| Indrawing | No indrawing | 2.68 | 2.33, 3.09 |
| Grunting | No grunting | 1.77 | 1.57, 2.00 |
| Crackles | No crackles | 1.18 | 1.06, 1.32 |
| Difficulty drinking | No difficulty drinking | 1.39 | 1.21, 1.59 |
| Pallor | No pallor | 1.44 | 1.22, 1.70 |
| Not alert | Alert | 1.55 | 1.28, 1.87 |
| 2 days illness before admission | 1 day illness before admission | 0.90 | 0.77, 1.06 |
| 3 days illness before admission |  | 0.85 | 0.73, 1.00 |
| 4 days illness before admission |  | 0.80 | 0.65, 0.98 |
| 4+ days illness before admission |  | 0.83 | 0.72, 0.97 |
| Pneumonia | No pneumonia | 1.76 | 1.49, 2.07 |
| Malaria | No malaria | 0.83 | 0.68, 1.02 |
| Tuberculosis | No Tuberculosis | 0.94 | 0.69, 1.30 |
| Diarrhoea | No diarrhoea | 0.85 | 0.74, 0.97 |
| Dehydration | No dehydration | 0.97 | 0.82, 1.14 |
| Malnutrition | No malnutrition | 0.77 | 0.64, 0.92 |
| Anaemia | No anaemia | 1.27 | 1.01, 1.59 |
| Meningitis | No meningitis | 1.22 | 0.98, 1.52 |
| Asthma | No asthma | 1.94 | 1.59, 2.35 |
| Bronchiolitis | No bronchiolitis | 1.88 | 1.47, 2.41 |
| Sepsis | No sepsis | 1.56 | 1.09, 2.24 |
| Antimalarials - neither | Antimalarials - injectable | 1.33 | 1.07, 1.64 |
| Antimalarials - oral |  | 0.89 | 0.56, 1.39 |
