# Supplementary material for: Variability in the use of pulse oximeters with children in Kenyan hospitals: A mixed-methods analysis
Source: PLoS Med. 2019 Dec 31;16(12):e1002987. doi: 10.1371/journal.pmed.1002987 (PMC6938307; doi:10.1371/journal.pmed.1002987)
Supplement: S1 Text — MAR, Missing at Random. (DOCX) [file pmed.1002987.s005.docx]

S1 Text. Determining whether the data could be considered Missing At Random

To determine if the data could be considered Missing At Random (MAR) rather than Missing Not At Random (MNAR), and if so which variables influenced the likelihood of missing data, we investigated whether missingness was more likely for certain values of particular variables than for other values of these variables.

We created a variable for the number of missing variables for each child. These scores were averaged out to create a mean number of missing variables across the children. In addition to age and sex, which are routinely examined, we considered whether the data’s missingness levels were potentially influenced by the following variables, chosen based on topic knowledge and through speaking to people involved with data collection: admission month, weekend admission, admission time period (admission coded according to the 6-month interval in which the admission date occurred), hospital, Paediatric Admission Record (PAR) use, outcome. We also tested the pulse oximeter use and oxygen use variables as it may not be possible to run multiple imputation if the outcome variable influences the missing data levels.

To test which if any of these variables influenced the missing data levels, we first calculated the mean number of missing variables per child for each value of each variable (e.g. the mean number of missing variables of males vs. the mean number of missing variables of females); we graphed this relationship and then tested whether the difference between these values was significant (p<0.05), through Chi-squared analyses for the categorical variables, and regression for the age in years continuous variable. If a significant difference was found, this would indicate that the variable(s) were associated with the data’s missingness levels; the dataset’s missingness could then be considered MAR and the variable(s) in question would need to be included in the multiple imputation process, so that its/their influence could be taken into account in the prediction of the missing values.

According to the Chi-squared analyses and regression there was a significant association (p<0.05) between missingness levels and values of admission month, age, and PAR use, but no association for values of weekend admission, admission time period, hospital, sex, outcome, pulse oximeter use or oxygen use.

Therefore, as we had identified factors that could help explain the missing data levels and patterns, it is likely that the data were MAR. We therefore included the admission month, age, and PAR use variables in the multiple imputation process, to ensure that their effect on missingness levels was taken into account in creating estimates for the missing values.[1]

Then, after carrying out the multiple imputation process we checked for bias in the imputed data due to violation of the MAR assumption. Based on the following analyses we concluded that the multiple imputation process accurately retained the existing data’s characteristics in estimating the missing values:

i) Comparison of original data vs. imputed data

The means and variances of the continuous variables age and weight-for-age, and the proportions of PAR use, very high respiratory rate, and capillary refill (the 3 categorical variables with the largest amount of missing data) were essentially constant across each of the 30 imputed datasets. Box plots and density plots of age and weight-for-age values showed close similarities between the original dataset and the imputed datasets.

ii) Comparison of complete case data from the original dataset vs. imputed data

The imputed data summary statistics differed from the complete case summary statistics for admission time period, PAR use and pulse oximeter use, but were similar to those of the original dataset, which is to be expected because the children with missing data also differed from the complete case children for these variables and the imputation process aims to maintain the original dataset’s characteristics. The imputed data summary statistics were similar to the complete case summary statistics for all other variables. Although there were some differences between the complete case data and imputed data, given that this was a comparison of 10,000 vs. 800,000 measurements, across 45 variables, it is remarkable how little variation there was.

Finally, there were some differences between the coefficients and confidence intervals produced when using complete cases vs. the imputed datasets. Some differences were to be expected, given that the imputation process maintains the original dataset’s characteristics (and the characteristics of the original dataset’s complete cases differed from the characteristics of the overall original dataset, because of differences between children with vs. without missing data). However, these differences were not substantial enough to warrant concern as each confidence interval of the complete cases overlapped with the corresponding imputed data’s confidence interval.

References:

1. Sterne JAC, White IR, Carlin JB, Spratt M, Royston P, Kenward MG, et al. Multiple imputation for missing data in epidemiological and clinical research: potential and pitfalls. BMJ 2009; 338: b2393.
