# Supplementary material for: Variability in the use of pulse oximeters with children in Kenyan hospitals: A mixed-methods analysis
Source: PLoS Med. 2019 Dec 31;16(12):e1002987. doi: 10.1371/journal.pmed.1002987 (PMC6938307; doi:10.1371/journal.pmed.1002987)
Supplement: S2 Text — (DOCX) [file pmed.1002987.s006.docx]

S2 Text. Developing a best-fit logistic regression model

We applied the following methods to produce a best-fit logistic regression model that examined (i) with which children pulse oximeters are used, and (ii) the factors that influence whether oxygen is prescribed.

We compared the output from the two methods as a form of sensitivity analysis to guide the selection of the final best-fit model.

i) Reduced Model A

We manually derived Reduced Model A by using backwards stepwise regression.

The process for backwards stepwise regression was as follows: we first removed the non-statistically significant variable (p≥0.05) with the smallest coefficient from the model, with the exception of the control variables. If the coefficients and p-values of the remaining variables did not change substantially we excluded the variable from the model and continued to remove non-statistically significant variables with the next smallest coefficient. We continued this process, removing one variable at a time until the only variables that remained were the control variables, variables that contributed significantly (p<0.05), or variables that led to a substantial change in the model’s output when removed. ‘Substantial change’ was a subjective measure; with each iteration of the regression, a coefficient of 0.184 may change to e.g. 0.189 without causing concern, but if after a variable was removed it changed to e.g. 0.350, and various other variables underwent similar changes, this was judged to be a substantial change and the variable was retained in the model. We did not remove any interaction terms until both of the individual component variables of the interaction term had been removed.

i) Reduced Model B

We carried out the following steps to produce a Reduced Model B:

- We randomly selected 3 out of the 30 imputed datasets that had been created when multiple imputation was carried out with the original dataset
- We used bootstrapping to create 30 datasets from each of these 3 imputed datasets
- We carried out stepwise selection using Akaike information criterion (AIC) on each of the 3 collections of 30 datasets; this produced 30x3=90 reduced models
- Independently from the above steps, we ran stepwise selection using AIC for each of the 30 imputed datasets created from the original dataset through multiple imputation; this produced 30 reduced models
- Finally, we selected the variables that were found in 50% or more of these 120 AIC models, and included these variables to create Reduced Model B

S3 Text Fig 1 shows this process.

S3 Text Fig 1: Process to create Reduced Model B


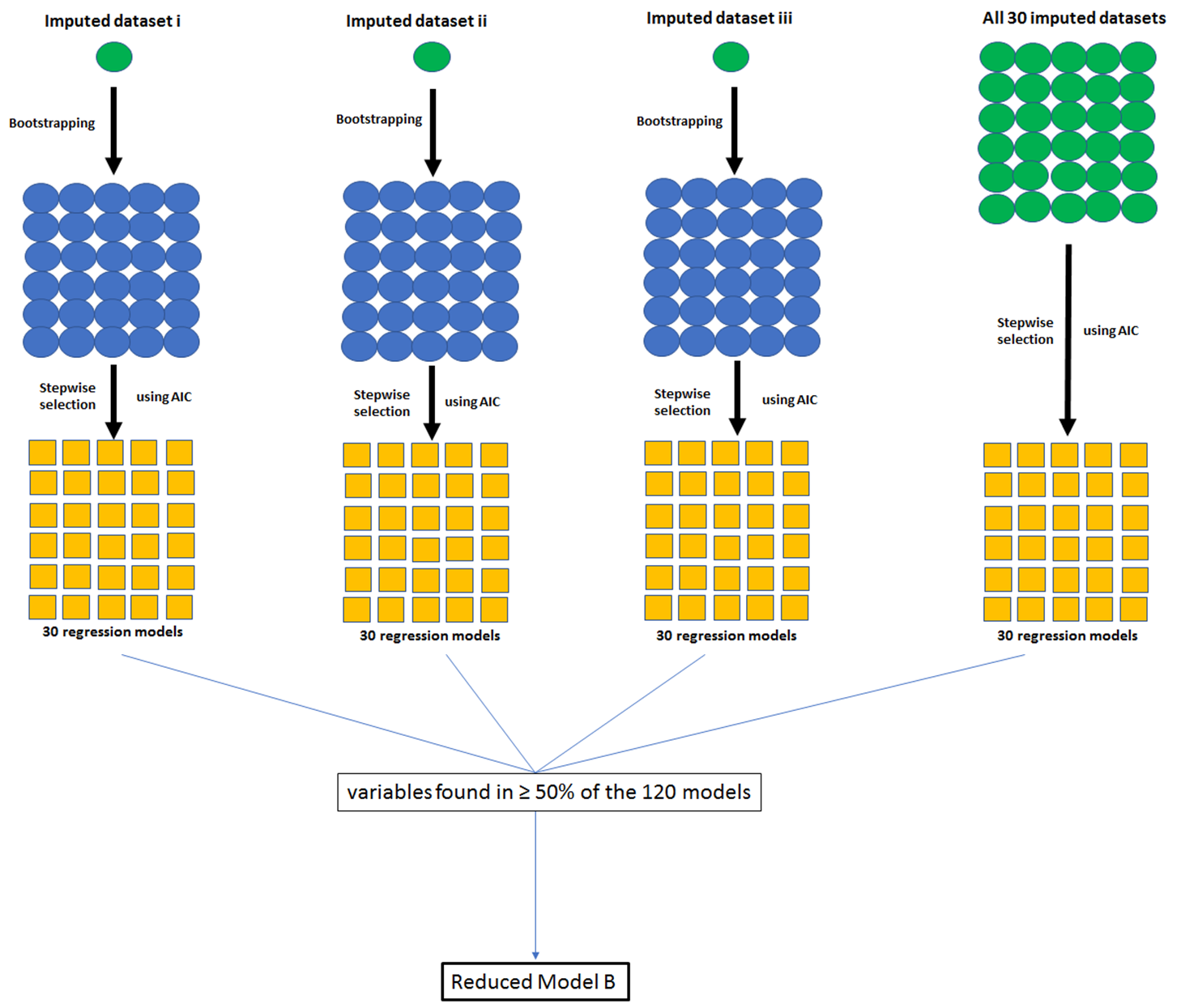


Reduced Model B included variables found in the majority of the 120 models produced through stepwise selection using AIC on i) 3x30 models produced through bootstrapping of 3 randomly chosen multiply imputed datasets, and ii) all 30 datasets originally produced through multiple imputation

Finally, we compared Reduced Model A and Reduced Model B to decide which was the best-fit model, based on topic knowledge, and the odds ratios and confidence intervals produced by each model

When we compared the models we saw that all of the variables included in the manual model (Model A) were also included in the AIC model (Model B) but the AIC model included additional variables. However, when these models were separately run with each of the 30 imputed datasets and the results were pooled using Rubin’s rules, the odds ratios and confidence intervals of the variables included in both models were almost exactly the same, and none of the variables that were only included in the AIC model were statistically significant (based on confidence interval spread). The results we present are therefore from the manual model. See S3 Text Table 1 for the full list of variables included in the full model from which the best-fit model was produced.

S3 Text Table 1. Variables included in the logistic regressions to identify (i) with which children pulse oximeters are used, and (ii) the association of pulse oximeter use on subsequent oxygen therapy

| **Variable** | **Included in the full model from which the best-fit model was produced for the regression examining (i) pulse oximeter use and/or (ii) oxygen therapy** | **Why it was included in the full model for the regression(s)** |
| --- | --- | --- |
| *Pulse oximeter use* | *i** | Outcome of interest |
| *Oxygen use* | *ii** | Outcome of interest |
| *Weekend admission* | *i* & ii** | Explanatory variable: Care may be reduced on a weekend when there are fewer staff available |
| *Admission time period* | *i* & ii** | Control variable: Pulse oximeter use could have changed over time because of general improvements over time, events such as guideline introductions, and the build-up of CIN feedback |
| *Hospital* | *i* & ii** | Control variable: Varying hospital characteristics e.g. concerning resource availability, management structures and staffing, differences in illness and socioeconomic profiles of patients etc. could cause differences in care |
| *Sex* | *i* & ii** | Control variable: Pulse oximeters might be used more commonly with males or females |
| *Hospital use of pulse oximeters by admission month* | *i* & ii** | Control variable: The reasons for pulse oximeter use may be different depending on whether at that hospital at that time, pulse oximeters are used commonly or not. |
| *Age in years* | *i* & ii** | Control variable: Younger children may be seen as more vulnerable and so in greater need of a pulse oximeter reading |
| *Weight-for-age* | *i* & ii** | Control variable: An indication for malnutrition; could impact HCWs’ views on diagnosis and illness severity |
| *Paediatric Admission Record (PAR) use^i^* | *i* & ii** | Control variable: HCWs might stick to guidelines more closely when using the PAR |
| *Length of illness before admission* | *i & ii** | Explanatory variable: An indication of illness severity |
| *Fever* | *i* & ii** | Explanatory variable: A common symptom |
| *Cough* | *i* & ii** | Explanatory variable: A respiratory symptom |
| *Difficulty breathing* | *i* & ii** | Explanatory variable: The evidence suggests this is one of the best clinical signs for indicating hypoxemia [1] |
| *Vomit everything* | *i* & ii** | Explanatory variable:  -Indicates severity of illness [2] (could prompt pulse oximeter use)  -Non-respiratory symptom associated with severe illness (could discourage pulse oximeter use) |
| *Difficulty feeding* | *i & ii** | Explanatory variable:  -An indication of illness severity  -The evidence suggests this is one of the best clinical signs for indicating hypoxemia [1] |
| *Convulsions* | *i & ii** | Explanatory variable: A sign of illness severity [2,3] |
| *Very high respiratory rate* | *i* & ii** | Explanatory variable:  -An indication of respiratory illness severity  -The evidence suggests this is one of the best clinical signs for indicating hypoxemia [4] |
| *Oedema* | *i & ii* | Explanatory variable: Indicates severe acute malnutrition [2] |
| *Stridor* | *i & ii* | Explanatory variable: Indicates severe pneumonia [2] |
| *Central cyanosis* | *i & ii** | Explanatory variable:  -Indicates severity of illness [3,5]  -An indication of respiratory illness severity  -The evidence suggests this is one of the best clinical signs for indicating hypoxemia [1,6] |
| *Chest indrawing* | *i* & ii** | Explanatory variable:  - Indicates severity of respiratory illness  -The evidence suggests this is one of the best clinical signs for indicating hypoxemia [1,6] |
| *Grunting* | *i & ii** | Explanatory variable:  -Indicates severity of respiratory illness  -The evidence suggests this is one of the best clinical signs for indicating hypoxemia [1,6] |
| *Wheeze* | *i & ii* | Explanatory variable: Respiratory symptom |
| *Crackles* | *i & ii** | Explanatory variable:  -The evidence suggests this is one of the best clinical signs for indicating hypoxemia [4] |
| *Capillary refill* | *i & ii* | Explanatory variable: A sign of illness severity [3,5] |
| *Pallor* | *i & ii** | Explanatory variable: A sign that is indicative of anaemia [2] |
| *Lack of alertness* | *i* & ii** | Explanatory variable:  -A sign of illness severity [2,3]  -The evidence suggests this is one of the best clinical signs for indicating hypoxemia [4] |
| *Difficulty drinking* | *i* & ii** | Explanatory variable:  -General sign of severity of illness [2]  -The evidence suggests this is one of the best clinical signs for indicating hypoxemia [1] |
| *Stiff neck* | *i & ii* | Explanatory variable:  -A sign that is indicative of severe malaria or other severe febrile disease [2] |
| *Bulging fontanelle* | *i & ii* | Explanatory variable:  -A sign that is indicative of meningitis [5] |
| The 15 2-way combinations between *Difficulty feeding, Very high respiratory rate, Central cyanosis, Chest indrawing, Grunting, and Lack of alertness* | *i* & ii (if significant in best fit regression i)* | Explanatory variables:  -These symptoms are all indicators of illness severity  -It is likely that HCWs might base care decisions on combinations of symptoms rather than based on just one symptom |
| 11 diagnoses variables: pneumonia, malaria, TB, diarrhoea, dehydration, malnutrition, anaemia, meningitis, asthma, bronchiolitis, sepsis | ii* | Control variables: HCWs might follow guidelines on oxygen provision for particular diagnoses |
| Antimalarials | ii* | Control variable: a marker of severity |

* = included in best-fit model

Note: To determine whether children were more likely to be prescribed oxygen if they had a pulse oximeter reading below 90% than if they had a pulse oximeter reading of 90% or above, we replaced the variable ‘pulse oximeter was used’ with the variable ‘value of the pulse oximeter reading’ in the model examining the factors that influence whether oxygen is prescribed. This was to control for all variables other than pulse oximeter use that might influence oxygen provision, to determine how HCWs’ decision to use oxygen is determined by a high vs. low pulse oximeter value.

References

1. Basnet S, Adhikari RK, Gurung CK. Hypoxemia in Children with Pneumonia and Its Clinical Predictors. Indian Journal of Pediatrics 2006;73(9):777-781.

2. World Health Organization. Integrated Management of Childhood Illness: Chart Booklet. 2014.

3. Kenyan Ministry of Health. Basic Paediatric Protocols for ages up to 5 years. 2016;4th edition.

4. Zhang L, Mendoza-Sassi R, Santos JC, Lau J. Accuracy of symptoms and signs in predicting hypoxaemia among young children with acute respiratory infection: a meta-analysis. International Journal of Tuberculosis and Lung Disease 2011;15(3):317-325.

5. World Health Organization. Pocket book of hospital care for children: guidelines for the management of common childhood illnesses. 2013;2nd edition.

6. Wandeler G, Pauchard JY, Zangger E, Diawara H, Gehri M. Which clinical signs predict hypoxaemia in young Senegalese children with acute lower respiratory tract disease?. Paediatrics and international child health 2015;35(1):65-68.
