# Supplementary material for: Variability in the use of pulse oximeters with children in Kenyan hospitals: A mixed-methods analysis
Source: PLoS Med. 2019 Dec 31;16(12):e1002987. doi: 10.1371/journal.pmed.1002987 (PMC6938307; doi:10.1371/journal.pmed.1002987)
Supplement: S3 Text — (DOCX) [file pmed.1002987.s007.docx]

S3 Text. Semi-structured interview topic guide

(bold indicates those questions to focus on)

**1. Can you tell me a bit about your role in the process of admitting children to the hospital, and what sort of tasks you do on an average day?**

**2. How do you try to figure out the diagnosis and disease severity of children admitted to your hospital?**

a) Do you use any diagnostic tools to help with this?

b) How do you choose which tool to use?

**3. How do you decide when to give oxygen to a child?**

a) Which symptoms do you think suggest that a child needs oxygen?

b) Do you use any diagnostic tools to help decide whether to give oxygen?

**c) *if use PO:* What oxygen saturation value would you use to decide whether to give oxygen?**

d) *if use 90% threshold*: **Would you ever give oxygen to a child who has an oxygen saturation higher than 90%? // What would you do if a child has an oxygen saturation above 90% but their symptoms suggest that they need oxygen? Would you give them oxygen?**

e) *if use 90% threshold:* **Are there times when you wouldn’t give oxygen to a child even if their oxygen saturation value was less than 90%? // What would you do if a child has an oxygen saturation below 90% but their symptoms don’t seem to suggest that they need oxygen? Would you give them oxygen?**

**4. What do you think of pulse oximeters? For instance, how useful do you think they are?**

**a) Which children are they most useful for at admission?**

**b) Are there any children who you think don’t need a pulse oximeter reading at admission?**

**c) How reliable do you think they are?**

**d) How easy to use are they?**

**i) Are there ever times when you have difficulty using them?**

**5. Are there ever any situations when you want to use a pulse oximeter but you’re not able to for some reason?**

**a) How often does this happen?**

b) What do you think could help to solve this problem?

**c) What do you do if you don’t have a pulse oximeter?**

d) Could you please tell me about any other equipment that you have similar issues with?

*6. If too busy / too many patients / not enough pulse oximeters is brought up then can ask the following additional follow up question:*

***a) How do you decide which children to use the pulse oximeter with?***

**7. How many pulse oximeters do you have in the ward?**

**a) What kind are those? [Monitor? Battery-operated? Handheld?]**

**b) Do you think this is enough pulse oximeters for the ward?**

**c) How many do you think would be a good number to have in the ward?**

**8. How often do the pulse oximeters break?**

**a) What happens when a pulse oximeter breaks? What’s the process for repairing it?**

b) [*If sent somewhere*]: where is it sent?

**c) How long does it take before the pulse oximeter is fixed?**

d) Who is responsible for dealing with a broken pulse oximeter?

e) On average, how many pulse oximeters are working on any given day?

**9. How often do pulse oximeter batteries run out?**

**a) How are they replaced?**

**b) How long does that take?**

**10.** *If at a hospital where pulse oximeters used between 9/2013 and 2/2016:* **Here is a graph showing the % of children who obtained a pulse oximeter reading at admission at your hospital each month from September 2013 through February 2016.**

**a) Why do you think there is so much variability? // Why do you think pulse oximeters are used more often in some months than in others?**

b) Why do you think that pulse oximeter use increased overall over this time period?

**11.** *If at a hospital where pulse oximeters were not used between 9/2013 and 2/2016:* **We looked at pulse oximeter use at admission at different hospitals in Kenya and we found that there was a lot of variability: pulse oximeters were used a lot more often in some months than in others, and this was different for different hospitals**

**a) Why do you think there is so much variability? // Why do you think pulse oximeters are used more often in some months than in others?**

**12. What’s the process if you or someone else in the ward wants to request a new piece of equipment?**

**a) How long does that take?**

**b) Who is responsible for doing that?**

**c) Have you gone through this process?**

**13. When did you first hear about pulse oximetry?**

a) What form of training, if any, was provided then?

b) What did you learn about pulse oximetry then?

14. How was pulse oximetry introduced in your hospital?

a) Was any training provided then?

**15. What sort of training on pulse oximeters, if any, has there been since you have been working in this ward?**

**a) Do you think that more training is necessary?**

**b) How do you think it would be best for this training to be carried out?**

**16. Can you please tell me about oxygen availability at your hospital?**

a) Can you give me any examples of times when you have wanted to give oxygen to a child but have not been able to for some reason?

b) What do you do if you don’t have enough oxygen?

**17. Roughly what proportion of children do you give oxygen to?**

**a) What proportion of children with pneumonia do you give oxygen to?**

**18. When you use a pulse oximeter or give oxygen, where do you record that information?**

**19. You’ve mentioned about how pulse oximeters affect your decision of whether to give oxygen or not; do pulse oximeters also affect you decision of whether to give other treatments, for instance antibiotics?**

**20. Do you think that there is any difference in care based on the time of day or day of the week, for instance in the day vs. the night, or on weekends vs. weekdays?**

**21. Finally, what do you think could be done to encourage health workers in your ward to use pulse oximeters with children more often?**
