# Supplementary material for: Variability in the use of pulse oximeters with children in Kenyan hospitals: A mixed-methods analysis
Source: PLoS Med. 2019 Dec 31;16(12):e1002987. doi: 10.1371/journal.pmed.1002987 (PMC6938307; doi:10.1371/journal.pmed.1002987)
Supplement: S1 Study Protocol — (DOCX) [file pmed.1002987.s008.docx]

S1 Study Protocol. Quantitative research protocol

**Aim**:

To determine the use of pulse oximeters in the management of children in Kenyan hospitals

**Background**:

Hypoxemia refers to inadequate levels of oxygen in the blood, and occurs in a range of situations in newborns, children and adolescents. It is a common symptom of lower respiratory diseases, such as pneumonia and bronchiolitis, as well as of asthma, sepsis, meningitis, congenital heart disease, and various other conditions. The presence of hypoxemia in newborns, children and adolescents with such diseases can indicate complications and an increased risk of death [1].

While pulse oximetry is an important medical tool for detecting hypoxemia, there is considerable uncertainty about the impact it has on patient health outcomes when used in clinical decision-making. The evidence supporting the use of pulse oximeters in the management of sick children is from non-controlled before-after studies that measure outcomes before and after pulse oximeters were introduced into hospitals or pulse oximeter recordings were told to physicians. While there is some evidence to suggest a reduction in mortality and other health outcomes, and a reduced time in the emergency department, the quality of evidence for these outcomes is poor [2].

A range of pulse oximeters are now available, including some designed specifically for the conditions expected in low income settings: thus some are low cost, robust, portable and battery operated, while versions have been developed to be used with smartphones [3,4].

However often pulse oximeters are not available in low income countries, and if they are available, they are often not used [5,6,7]. Thus Desalu et.al 2011 found that only 38% of tertiary hospitals in Nigeria had pulse oximeters, while English et.al. 2014 found that pulse oximeters were not available in any of 22 hospitals in Kenya until 2012 when three hospitals obtained this technology [6,8]. McCollum et.al. 2013 conducted a study in five hospitals in Malawi in 2011 and found that only 13% of Malawian children obtained a pulse oximeter recording at admission to one of the hospitals while no readings were obtained in four hospitals [9]. In a survey of hospital clinicians across Cambodia, Ginsburg et.al. 2014 found that about half of clinicians said they either never use pulse oximeters to help diagnose and manage childhood pneumonia or do so only a little bit of the time [10]. In a survey of 97 clinicians from 19 countries in Africa, Asia and South America, at least one pulse oximeter was available at 71% of health facilities and only 59% of clinicians said they had access to a pulse oximeter most or all of the time; however, only 18% said they used pulse oximeters for help in diagnosing childhood pneumonia most or all of the time, while 44% said they had never done so [11]. This issue is not limited to low income countries. The 2003 joint guidelines on asthma management of the British Thoracic Society and the Scottish Intercollegiate Guidelines Network recommend measuring oxygen saturation in primary and secondary care to help determine acute asthma severity; however, in Scotland, Cunningham et.al. 2006 found that only 4% of general practices in Edinburgh had pulse oximeters available, and oxygen saturation monitoring was only recorded in 2% of primary care referrals of children with acute wheezing from general practices to the emergency department [12].

Evidence from the literature indicates that pulse oximeter use may vary depending on characteristics of children such as age, respiratory symptoms, diagnosis, and date of admission. Accordingly, a national study on the quality of emergency department care in the US in treating pneumonia found that pulse oximeter use by physicians varied depending on the age of the patient [13]. Furthermore, in a study that interviewed nurses in a pediatric intensive care unit in the UK, only 45% and 10% thought that pulse oximetry was indicated in patients with respiratory distress and respiratory failure, respectively, showing that decisions to use pulse oximeters can vary depending on the respiratory symptoms [14]. Diagnoses can also affect pulse oximeter use: most of the Cambodian clinicians interviewed in Ginsburg et.al. 2014’s study thought that pulse oximeters are more useful for general care than for managing pneumonia care [10]. The date of admission can play a role too: Berkeley et.al. 2004 found that the mortality rate of children in a district hospital in Kenya was 6% for those admitted on the weekend and 5% for those admitted on weekdays; they argued that delays in identification and treatment of hypoxemia may have been a key factor in driving this disparity [15]. English et.al. 2009 also found differences in hospital care in Kenya on weekends vs. weekdays as children under five were treated in maternal and child health clinics if they presented on a weekday, but were treated at general outpatient or casualty departments if presenting in the evening or on the weekend [16].

To be able to tackle the issues limiting the implementation of pulse oximeters it is first necessary to understand in what situations pulse oximeters are used vs. not used and why, and the implications of pulse oximeter use. This would enable programs to be tailored to particular contexts, and resources to be utilized most effectively.

**Research questions:**

The following primary research questions will be examined:

1. How much missing data is there for each CIN dataset variable? What is the distribution of the missing data?

2. What are the characteristics of the children included in the CIN dataset?

3. How has pulse oximeter use changed over time at each of the hospitals included in the CIN dataset?

4. Pulse oximeters are more likely to be used for children with which characteristics?

Are children with a diagnosis of pneumonia or who present with danger signs indicative of serious illness (e.g. AVPU, convulsion, inability to drink) more likely to obtain pulse oximeter readings than is the general population of children?

5. Are children who get pulse oximeter readings more likely to obtain particular treatments (e.g. oxygen therapy, or specific antibiotics) than children who do not get pulse oximeter readings?

Are children who obtain pulse oximeter readings more likely to obtain particular treatments than children with similar characteristics (e.g. hospital, age, diagnosis, illness severity) who do not obtain pulse oximeter readings?

**Methods:**

Data collection

A Clinical Information Network (CIN) was developed to collect data on children aged 1 month to 12 years entering 14 hospitals in Kenya. The CIN was developed through a collaboration between the Kenya Medical Research Institute (KEMRI) Wellcome Trust Research Programme’s (KWTRP) Health Services Unit (HSU), the Kenyan Ministry of Health, and the Kenya Paediatric Association [17]. So far data on over 37,000 admissions have been collected, from September 2013, and data collection is on-going [17,18].

The following data from the CIN will be used: date of admission, date of discharge/death, hospital, age, weight, mid-upper arm circumference, gender, vaccination status, symptoms, symptoms indicative of serious illness (AVPU, convulsions, inability to drink), pulse oximeter use, pulse oximeter reading, number of times oxygen saturation monitored in 48 hours, use of other diagnostic tools, diagnoses, antibiotics and other drugs given, oxygen ordered, oxygen flow rate, oxygen route of admission, date of oxygen prescription, other treatments given, alive/dead at discharge, condition at discharge (normal, neuro sequelae/disability, other), referral, follow-up care. See Appendix 1 for table with complete list of CIN variables that will be examined.

Plan of analysis

For each question, children will be considered in two groups: those aged between 1 month and 59 months and children aged 1 month to 12 years.

**Research question 1: How much missing data is there for each CIN dataset variable? What is the distribution of the missing data?**

We will calculate for each variable the percentage of participants for whom there is missing data, and for how many variables each participant has missing data. We will consider the distribution of the missing data by investigating which variables have more missing data than others and if there are any patterns to the variables or participants that have missing data / have higher levels of missing data. We will produce a table showing the amount of missing data for each variable, by hospital and time (where time will be the number of the month that the patient presented to the hospital, with months numbered 1-24 from the start of data collection to the present).

We will need to consider how the extent and distribution of missing data will determine which variables and participants we can include in the analyses and how we will deal with the missing data. If >40% of participants have data missing for a particular variable then we may have to exclude that variable from the analyses. If we find that there is very little data available in the early months of data collection then we may need to begin analyses from later. Clearly we will need to exclude participants from hospitals in which no pulse oximetry is undertaken from the analyses of pulse oximeter change over time, and child characteristics that increase the likelihood of pulse oximeter use. We may need to alter some of the research questions because of the distribution of missing data; for instance if data are extensively missing for most of the child characteristic variables but there are good data for the children who present with a cough / difficulty breathing then we may need to focus instead on this subsection of the population.

We will then try to determine whether these data are likely to be missing for random or non-random reasons. This judgement will involve considering the relative level of missing data for variables within a range of categories to determine if a specific variable impacts the rate of missing data.

The first step for dealing with the missing data will be to consider combinations of variables to determine if people have similar variables that are missing/complete. From this information we will determine whether to use complete case or imputation to deal with the missing data.

If the complete case method of dealing with missing data is used, then only cases that have data for the variables that we want to investigate will be included in the analyses; however this may introduce bias because we would be assuming that the patients/variables that are missing are random, i.e. there is no consistent reason why the ones that are missing are missing. Alternatively we may use imputation, where we would predict the values of the missing data; bias associated with this method involves the fact that by predicting values from a smaller set of values we would be making the overall data less variable than it probably is in reality. If it is possible, complete case will be used, due to its more straightforward procedures and because the large size of the dataset (more than 37,000 admissions) reduces the likelihood of bias associated with this method.

However more complex methods of dealing with missing data may be used if necessary. Results from using different methods may be compared; similar results would suggest a lower risk of bias from the chosen missing data method(s), and higher chance of accuracy of the results.

**Research question 2: What are the characteristics of the children included in the CIN dataset?**

We will investigate the descriptive characteristics of the participants. This will involve calculating the number and percentage of participants who obtained pulse oximeter recordings, the number and percentage by reading value and by the number of recordings taken. We will also calculate the number and percentage of participants by age; gender; weight for age (calculated from weight and age); respiratory symptoms; other symptoms; danger signs indicative of severe illness (AVPU, ability to drink, convulsions); diagnosis; vaccination status; whether and how often temperature, vital signs, blood pressure and pulse were monitored; number admitted per hospital; date of admission (weekday vs. weekend; season); whether, how much and how oxygen was prescribed; other treatments; and outcomes. See Appendix 1 for a full list of characteristics. Furthermore we will calculate the number and percentage of participants with each of the above characteristics who obtained pulse oximeter recordings.

**Research question 3: How has pulse oximeter use changed over time at each of the hospitals included in the CIN dataset?**

We will describe (through graphical and written descriptions) how pulse oximeter use has changed over time at each individual hospital.

This will involve describing the number and percentage of participants by hospital who obtained pulse oximeter results each month from when the data began to be collected 12-24 months ago (depending on the hospital) until the present. Time will be considered in monthly intervals. We will look at changes over time in terms of changes between specific dates and also relative amount of time since data began to be collected from each specific hospital.

We will also investigate whether there is a pattern to the monthly rate of admissions (of all children, and of those with respiratory disease) at each hospital – this will enable us to see if any patterns in pulse oximeter use may be related to changes in admissions (due to e.g. cyclical or seasonal effects). If patterns in admissions are found then we will also describe (through graphical and written descriptions) how pulse oximeter use has been changing at each hospital between these periods (e.g. between high and low seasons).

**Research question 4: Pulse oximeters are more likely to be used for children with which characteristics?**

We will use logistic regression to determine if pulse oximeters are more or less likely to be used for children with particular characteristics. The characteristics which we will investigate are: age, weight, weight for age, gender, symptoms, danger signs indicative of severe illness, diagnoses and date of admission (weekday vs. weekend; season). See Appendix 1 for a list of the specific symptoms and diagnoses. The response variable will be if a pulse oximeter recording was obtained (binary, yes or no), and the explanatory variable will be the characteristic of interest (e.g. age, symptom, etc.). All of the characteristics will be categorical variables (continuous variables such as age and weight will be converted to groups). An odds ratio will be produced, indicating the odds that a pulse oximeter reading was taken given that the patient had a particular characteristic.

As part of the logistic regression we will control for the hospital and time as confounders. The time variable will consist of the month/year of the date that the patient presented to the hospital. If we find in research question 3 that there is a pattern of pulse oximeter use that follows from a pattern in admission rates then we will also/instead (depending on the pattern) control for time as classified by the periods of the pattern (e.g. high and low seasons for pneumonia admissions). Alternatively, depending on the results from research question 3, it may make sense to categorize months as those in which pulse oximeter use is high vs. low, and then control for time based on these categories. See regression section below for further details on regression methods.

We will also use logistic regression to determine if children with a diagnosis of pneumonia or who present with danger signs indicative of serious illness (e.g. AVPU, convulsion, inability to drink) are more likely to obtain pulse oximeter readings than the general population of children presenting to the hospitals. The response variable will be if a pulse oximeter recording was obtained (binary, yes or no), and the explanatory variable will be the group of interest, i.e. children with a pneumonia diagnosis, those presenting with danger signs, or all children. We will control for confounders as part of this logistic regression, specifically hospital, patient age, and time. Time will be classified in the same manner as that used in the earlier part of research question 4. See regression section below for further details on regression methods.

Only associational, not causal, links will be able to be found with this data as there was no controlled introduction of pulse oximeters into the settings and there are no control groups with which to compare.

**Research question 5: Are children who obtain pulse oximeter readings more likely to obtain particular treatments (e.g. oxygen therapy, or specific antibiotics) than children who do not obtain pulse oximeter readings?**

We will consider broadly whether children who obtain pulse oximeter readings are more likely to obtain particular treatments than children who do not obtain pulse oximeter readings. However, if a relationship is found between pulse oximeter use and treatment choice and we did not do any further analyses we would not know whether this was because the actual act of taking a pulse oximeter reading and seeing its results affects treatment choice, or because health workers are choosing to use pulse oximeters with specific children who are also more likely to get certain treatments than the type of children who the health workers choose not to use pulse oximeters with (e.g. perhaps health workers predominantly use pulse oximeters with children who have severe symptoms rather than mild symptoms; children with severe symptoms are also likely to get different treatments than children with mild symptoms, regardless of pulse oximeter use; so if we found a relationship between pulse oximeter use and treatment choice we would not know if this was because the health workers’ treatment choices were affected by the pulse oximeter results, or if the health workers were simply giving different treatments to the children who were more ill than to the less ill children).

It is also possible that pulse oximeter use has an impact on treatment choice but only for children who have particular pulse oximeter results. For instance, if health workers have difficulty recognizing severe forms of illness without a pulse oximeter then children who obtain very low oxygen saturation scores from pulse oximeters may be more likely to get certain treatments than children with similar other characteristics who do not obtain pulse oximeter results. In such a situation, children who obtain higher pulse oximeter results might obtain the same treatments as children with other similar characteristics who do not obtain pulse oximeter results.

In the analyses of this research question we will therefore take into account the results from research question 4 and the relative oxygen saturation values of those who obtain pulse oximeter readings. We will thus investigate whether children who obtain pulse oximeter readings (considered as a whole group, and within subgroups of SaO2 <85%, 85-89%, 90-94%, ≥95%) are more likely to obtain particular treatments than children with similar characteristics (for those characteristics shown in the results for research question 4 to impact likelihood of pulse oximeter use, as well as hospital and time) who do not obtain pulse oximeter readings. We will thus be able to gain a better understanding of the reasons for why health workers make decisions about treatments, and whether/how pulse oximeter use plays a role in this.

We will use logistic regression to accomplish this. Separate regressions will be run for each treatment; in each the response variable will be if the type of treatment (oxygen therapy, specific antibiotic, etc.) was given (i.e. a binary variable, yes or no), and the explanatory variable will be if a pulse oximeter recording was taken (also a binary variable, yes or no), or the oxygen saturation range (an interval variable). We will control for confounders as part of this regression, specifically for the characteristics found in the results of research question 4 to influence likelihood of pulse oximeter use, as well as hospital and time. Time will be classified in the same manner as that used in research question 4. See regression section below for further details on regression methods.

Only associational, not causal, links will be able to be found with this data as there was no controlled introduction of pulse oximeters into the settings and there are no control groups with which to compare.

**Further details on the regression methods:**

*Linear regression*

Linear regression involves producing an equation which describes how a dependent variable (the outcome or response) changes with an increase in the independent variable (the exposure). The graphical line of this equation is created through the least squares method in which the sum of the squared vertical distances of the data points to the line is minimized. The generalized linear regression equation is y=β_0_ + β_1_x where β_0_ and β_1_ are known as regression coefficients or beta-coefficients, β_0_ is the intercept and β_1_ is the slope; standard errors are calculated for β_0_ and β_1_ to measure their precision. The regression coefficients can be divided by their standard errors to produce t-statistics, and p-values can be produced from these t-statistics to indicate whether there is evidence to support the null hypothesis that the beta-coefficients have a value of 0 (which would occur if there was no association between the exposure and the outcome); confidence intervals are also calculated [19].

Linear regression is used for measuring one interval, ratio or dichotomous explanatory variable’s effect on one interval or ratio response variable. If we want to look at how multiple explanatory variables impact a single binary response variable, such as in Research Questions 4 and 5, then we would use logistic regression [20].

*Logistic regression*

Logistic regression measures the association between one or multiple exposures and a binary outcome. Logistic regression model equations are highly related to odds ratio calculations, and are fitted on a log scale (so the effects of exposures are thought to be multiplicative instead of additive). The equation will thus be: log odds of outcome = β_0_ + β_1_x_1_ + β_2_x_2_ + … + β_p_x_p_, where β is the regression coefficient of the x exposure (graphically β is the slope of the x exposure) and is effectively the log of the x exposure odds ratio, and β_0_ is the log odds of the baseline exposure (the baseline exposure is the exposure option which the other exposure options are compared against). The maximum likelihood approach is used for this model [19].

When an exposure only has two options (e.g. exposed and unexposed, male and female) then it is treated as a binary and one exposure option is categorized as 1 while the other is categorized as 0. When an exposure has more than two options (e.g. multiple age groups, diagnoses) then indicator variables need to be used: n-1 indicator variables are created (where n is the number of options for the exposure) and the value of each indicator variable is composed of a combination of 0s and 1s with a 1 for the specific exposure option that the indicator variable is associated with and a 0 for all other exposure options; there is no indicator variable associated with the baseline exposure option. These indicator variables are then inserted into the logistic regression model [19].

Log odds ratios of the outcome, indicating the odds of the response given the exposure, are produced for each exposure option; when the exposure options are categorical, these odds ratios are compared to the baseline variable [19].

Standard errors and confidence intervals are also calculated, as are z statistics. When the exposure is binary, the z statistics can be used (through a Wald test) to test the null hypothesis that there is no relationship between the exposure and response, while the p-values help to show if there is evidence to support the null hypothesis. If the exposure has more than two options then this method of hypothesis testing is not as useful, as such tests would just measure the null hypothesis that the exposure option has no effect on the response, compared with the baseline exposure. Instead, to test the null hypothesis that the exposure as a whole (not just one of its specific options) does not have an effect on the response, either a multi-parameter Wald test can be used, or else likelihood ratio tests can be used, which produce a likelihood ratio statistic, which follows a χ^2^ distribution; in doing so a p-value is produced that indicates the strength of the evidence for the null hypothesis that there is no relationship between the exposure and outcome. The equation for the likelihood ratio statistic is LRS=-2 x (L_exc_ – L_inc_), where L_exc_ is the model log likelihood while excluding the variables of interest, and L_inc_ is the model log likelihood while including the variables of interest [19].

When conducting a multivariable model where several exposures are being considered within the same model (e.g. age, gender and diagnosis), then one needs to consider the effect of each exposure while controlling for the other exposures. Thus to test a null hypothesis that age does not have an impact on a response when controlling for diagnosis, we would create a likelihood ratio statistic by comparing the log likelihood of a model in which only diagnosis is included, with the log likelihood of a model which looks at age too [19].

Logistic regression makes the following assumptions: i) only important variables should be included but all important variables should be included, ii) each observation must be independent, iii) little or no multicollinearity, iv) there is a linear relationship between the independent variables and the log odds, v) the sample size needs to be large (at least 10-30 cases per independent variable). We will test for these assumptions. Thus we will ensure that the important, and only the important, variables are included by using a stepwise approach to the regression; and we know that the observations are independent because the cases are children who are separately presenting to hospital – it is not a before-after or paired design. To test that there is little or no multicollinearity we will compute the Pearson’s Bivariate Correlation matrix of the independent variables to ensure the correlation coefficients are less than 1; calculate Tolerance from an initial linear regression (a T <0.1 indicates possible multicollinearity and T <0.01 strongly suggests multicollinearity); calculate the Variance Inflation Factor (VIF = 1/T; VIF>10 indicates possible multicollinearity and VIF>100 strongly suggests linear regression); and determine a condition index by calculating a factor analysis with the independent variables (a condition index of 10-30 indicates possible multicollinearity and a condition index of >30 strongly suggests multicollinearity). The linear relationship between the independent variables and log odds will be ascertained by plotting the independent variables against the log odds; and we know the sample size will be large enough because even though there may be substantial amounts of missing data, data has been collected from over 37,000 children [20].

We will check that variables which we believe to be confounders are indeed confounders by comparing the adjusted and unadjusted (for the potential confounder) coefficient estimates for the explanatory variable(s); if the estimates differ by 10% or more then the variable will be considered a confounder.

**Further research:**

The results for the above research questions will then help to inform the next stages of research.

In the next stages it is likely that only children aged 1 month to 59 months will be considered as it is these children for whom health guidelines used in Kenya focus [21].

Missing data

Information on the level of missing data for each variable will enable us to determine what further analyses are possible. Knowing which hospitals have not had any or only very minimal pulse oximeter use will indicate which hospitals will need to be excluded from further analyses.

Information on the level of missing data for each variable, and whether this is missing for random or non-random reasons will also help us to determine which methods to use to deal with the missing data in any further analyses, including whether to explore more complex methods than those used in the first stage of analyses.

Treatment and outcome pathways

Further analyses are also likely to involve investigating how pulse oximeter use affects children’s treatment and outcome pathways: if children who obtain pulse oximeter recordings are more or less likely to obtain oxygen therapy, specific antibiotics or other treatments then their outcomes (mortality, morbidity) and/or length of hospital stay may also be affected. The results from the above research questions will help us to determine how to analyse how pulse oximeter use may affect these pathways, including what pathways to consider, and which statistical methods (e.g. regression, complex modelling) to use.

Qualitative research

In addition, we will use the results to develop further research questions to be investigated through qualitative methods such as interviews and focus groups with health workers, researchers, and other key informants in Kenyan hospitals. Potential questions could include: What are the barriers to pulse oximeter use? Why do health workers decide to use pulse oximeters with children of particular characteristics more so than with other children? Why do health workers at some hospitals use pulse oximeters while health workers at other hospitals do not? How and why did pulse oximeter use change after specific intervention events? How have health workers tried to increase pulse oximeter use?

**Ethical approval and confidentiality**

Ethical approval for the collection of the CIN data was granted by the Kenya Medical Research Institute (SSC 2465). The need for consent was waived. Oxford’s CUREC ethical group confirmed that no further ethical approval was needed for the project.

The CIN data are anonymized. Further measures will be taken to ensure the data remains secure and confidential, and that they are periodically backed up to encrypted USB drives and/or Oxford’s TSM backup service; particularly sensitive data will be stored on and analysed solely in Oxford’s secure High Compliance System. A separate Data Management Plan has been developed to discuss these issues and plans.

References

1. World Health Organization. Manual on use of oxygen therapy in children. 2014.

2. Enoch AJ, English M, Shepperd S. Does pulse oximeter use impact health outcomes? A systematic review. Archives of Disease in Childhood 2015;101:694-700.

3. Lifebox. Value of a Lifebox. 2015; Available from: <http://www.lifebox.org/safe-surgery/value-of-a-lifebox/>.

4. Peterson CL, Chen TP, Ansermino M, Dumont GA. Design and evaluation of a low-cost smartphone pulse oximeter. Sensors 2013;13(12):16882-16893.

5. World Health Organization. Global pulse oximetry project: first international consultation meeting. Background document. 2008.

6. English M, Gathara D, Mwinga S, Ayieko P, Opondo C, Aluvaala J, et al. Adoption of recommended practices and basic technologies in a low-income setting. Archives of Disease in Children 2014;99:452-456.

7. Walker IA, Merry AF, Wilson IH, McHugh GA, O’Sullivan E, Thoms GM, et al. Global oximetry: An international anaesthesia quality improvement project. Anaesthesia 2009;64(10): 1051-1060.

8. Desalu OO, Onyedum CC, Iseh KR, Salawu FK, Salami AK. Asthma in Nigeria: Are the facilities and resources available to support internationally endorsed standards of care? Health Policy 2011;99: 250-254.

9. McCollum ED, Bjornstad E, Preidis GA, Hosseinipour MC, Lufesi N. Multicenter study of hypoxemia prevalence and quality of oxygen treatment for hospitalized Malawian children. Transactions of the Royal Society of Tropical Medicine and Hygiene 2013;107(5):285-92.

10. Ginsburg AS, Gerth-Guyette E, Mollis B, Gardner M, Chham S. Oxygen and pulse oximetry in childhood pneumonia: surveys of clinicians and student clinicians in Cambodia. Tropical Medicine and International Health 2014;19(5):537-544.

11. Ginsburg AS, Van Cleve WC, Thompson MIW, English M. Oxygen and Pulse Oximetry in Childhood Pneumonia: A Survey of Healthcare Providers in Resource-limited Settings. Journal of tropical pediatrics 2012;58(5):389-393.

12. Cunningham S, McMurray A. Adoption of recommended practices and basic technologies in a low-income setting. Primary Care Respiratory Journal 2006;15: 98-101.

13. Pham JC, Kelen GD, Pronovost PJ. National study on the quality of emergency department care in the treatment of acute myocardial infarction and pneumonia. Academic emergency medicine : official journal of the Society for Academic Emergency Medicine 2007;14(10):856-863.

14. Jones M. The Oxygen Tissue Saturation (SPO2) Audit. Paediatric Research 2010;68:239.

15. Berkley JA, Brent A, Mwangi I, English M, Maitland K, Marsh K, et al. Mortality among Kenyan children admitted to a rural district hospital on weekends as compared with weekdays. Pediatrics 2004;114(6):1737-1738.

16. English M, Ntoburi S, Wagai J, Mbindyo P, Opiyo N, Ayieko P, et al. An intervention to improve paediatric and newborn care in Kenyan district hospitals: understanding the context. Implementation Science 2009;4:42.

17. Tuti T, Bitok M, Paton C, Makone B, Malla L, Muinga N, et al. Innovating to enhance clinical data management using non-commercial and open source solutions across a multi-center network supporting inpatient pediatric care and research in Kenya. Journal of the American Medical Informatics Association 2016;23(1):184-192.

18. Gathara D, Malla L, Ayieko P, Karuri S, Nyamai R, Irimu G, et al. Variation in and risk factors for paediatric inpatient all-cause mortality in a low income setting:  data from an emerging clinical information network. BMC Pediatrics 2017;17: 99

19 Kirkwood BR, Sterne JAC. Essential Medical Statistics. 2nd ed.: Blackwell; 2003.

20. Statistics Solutions. Assumptions of logistic regression. 2015; Available at: <https://www.statisticssolutions.com/assumptions-of-logistic-regression/>.

21. Kenyan Ministry of Health. Basic Paediatric Protocols for ages up to 5 years. 2016;4th edition.

Appendix 1:

CIN variables that will be used

| **Variable** | **Question in which variable is used** |
| --- | --- |
| Date of admission | 1, 2, 3, 4, 5 |
| Date of discharge/death | 1, 2 |
| Hospital name | 1, 2, 3, 4, 5 |
| Age (years + months + <1 month) | 1, 2, 4 |
| Weight | 1, 2, 4 |
| MUAC | 1, 2, 4 |
| Gender | 1, 2, 4 |
| Vaccination status | 1, 2, 4 |
| Length of illness | 1, 2, 4 |
| Fever | 1, 2, 4 |
| Fever duration | 1, 2, 4 |
| Cough | 1, 2, 4 |
| Cough duration | 1, 2, 4 |
| Cough > 2 weeks | 1, 2, 4 |
| Difficulty breathing | 1, 2, 4 |
| Diarrhoea | 1, 2, 4 |
| Vomiting everything | 1, 2, 4 |
| Difficulty feeding | 1, 2, 4 |
| Convulsions | 1, 2, 4 |
| Number of fits | 1, 2, 4 |
| Partial/focal fits | 1, 2, 4 |
| Temperature | 1, 2, 4 |
| Respiratory rate | 1, 2, 4 |
| PO used | 1, 2, 3, 4, 5 |
| PO reading | 1, 2, 5 |
| Blood pressure measured | 1, 2 |
| Thrush | 1, 2, 4 |
| Lymph nodes >1cm | 1, 2, 4 |
| Wrist/rib signs for rickets | 1, 2, 4 |
| Visible severe wasting | 1, 2, 4 |
| Oedema or Kwashiorkor | 1, 2, 4 |
| Stridor | 1, 2, 4 |
| Central cyanosis | 1, 2, 4 |
| Indrawing | 1, 2, 4 |
| Grunting | 1, 2, 4 |
| Acidotic breathing | 1, 2, 4 |
| Wheeze | 1, 2, 4 |
| Crackles/crepitations | 1, 2, 4 |
| CAP refill | 1, 2, 4 |
| Extremities warm up to | 1, 2, 4 |
| Pallor/anaemia | 1, 2, 4 |
| Sunken eyes | 1, 2, 4 |
| Skin pinch | 1, 2, 4 |
| AVPU | 1, 2, 4 |
| Ability to drink/breastfeed | 1, 2, 4 |
| Reduced movement/tone | 1, 2, 4 |
| Malaria test results | 1, 2, 4 |
| Primary admission diagnosis (malaria, severe malaria, non-severe malaria, non-classified malaria, pneumonia, diarrhoea/acute GE, dehydration, malnutrition, anaemia, meningitis, asthma, TB) | 1, 2, 4 |
| Primary admission diagnosis (written in) | 1, 2, 4 |
| Secondary admission diagnosis (malaria, severe malaria, non-severe malaria, non-classified malaria, pneumonia, diarrhoea/acute GE, dehydration, malnutrition, anaemia, meningitis, asthma, TB) | 1, 2, 4 |
| Secondary admission diagnosis (written in) | 1, 2, 4 |
| Antibiotics given including dose, frequency, duration (XPen, Gentamicin, Amoxicillin, Quinine, Artesunate) | 1, 2, 5 |
| Antibiotics and other drugs given (Ceftriaxone, CAF, Metronidazole, Cotrimoxazole, Anti-TBs, Anti-Malarials, Artemether, Coartem, Salbutamol, Predinsalone) | 1, 2, 5 |
| Oxygen ordered | 1, 2, 5 |
| Oxygen flow rate | 1, 2, 5 |
| Oxygen route of admission | 1, 2, 5 |
| Date oxygen prescribed | 1, 2, 5 |
| Other treatments given (10% dextrose bolus, blood transfusion, fluid bolus, fluid at admission for dehydration, IV fluids for dehydration, oral fluids, maintenance fluids, feeds at admission, feeds at other times) | 1, 2, 5 |
| Vital signs monitored in first 48 hours | 1, 2 |
| Number of times temperature monitored in 48 hours | 1, 2 |
| Number of times respiratory rate monitored in 48 hours | 1, 2 |
| Number of times pulse monitored in 48 hours | 1, 2 |
| Blood pressure monitored | 1, 2 |
| Oxygen Saturation monitored | 1, 2, 3, 4, 5 |
| Number of times oxygen saturation monitored in 48 hours | 1, 2, 3, 4, 5 |
| Alive or dead at discharge | 1, 2 |
| Whether discharged, referred, absconded, discharged against advice | 1, 2 |
| Condition on discharge: normal, neuro sequelae/disability, other | 1, 2 |
| Follow up care: none, hospital clinics, other health facilities | 1, 2, 5 |
| Primary diagnosis at discharge (malaria, severe malaria, non-severe malaria, non-classified malaria, pneumonia, diarrhoea/acute GE, dehydration, malnutrition, anaemia, meningitis, asthma, TB) | 1, 2 |
| Primary diagnosis at discharge (written in) | 1, 2 |
| Secondary diagnosis at discharge (malaria, severe malaria, non-severe malaria, non-classified malaria, pneumonia, diarrhoea/acute GE, dehydration, malnutrition, anaemia, meningitis, asthma, TB) | 1, 2 |
| Secondary diagnosis at discharge (written in) | 1, 2 |
| Discharge treatment (written in) | 1, 2, 5 |

Black = time; brown = child characteristics; red = symptoms; blue = diagnoses; dark green = treatments; orange = outcomes; purple = pulse oximetry; light green = oxygen therapy; yellow = other diagnostic/monitoring tools; grey = other
