# Supplementary material for: Variability in the use of pulse oximeters with children in Kenyan hospitals: A mixed-methods analysis
Source: PLoS Med. 2019 Dec 31;16(12):e1002987. doi: 10.1371/journal.pmed.1002987 (PMC6938307; doi:10.1371/journal.pmed.1002987)
Supplement: S2 Study Protocol — (DOCX) [file pmed.1002987.s009.docx]

S2 Study Protocol. Qualitative research protocol.

**Pulse oximeter use in Kenyan hospitals study protocol**

**Lay Summary**

**Pulse oximeter use in Kenyan hospitals: a qualitative study of health workers’ perspectives of the barriers to pulse oximeter use**

**Background**

Hypoxemia (low levels of oxygen in the blood) is associated with increased risk of death in newborns, children and adolescents, and is a common complication of bronchiolitis, pneumonia, asthma and other serious conditions (e.g. sepsis). Pulse oximeters are a low-cost tool that measure oxygen levels in the blood and so their use could lead to reductions in child mortality by helping to detect hypoxemia and increase the chance of prompt, effective treatment.

However, pulse oximeters are often not available, particularly in low-income settings and even when they are available, they are often not used, but little research in any setting has examined why this is the case.

**What questions are we trying to answer?**

From the perspective of health workers in Kenyan hospitals:

a) Why might health workers in Kenyan hospitals not have access to pulse oximeters?

b) In what situations might they choose not to use pulse oximeters with admitted children?

c) In what situations might a lack of pulse oximeter use, in an environment where pulse oximeters have been adopted, be due to structural/logistical factors (e.g. pulse oximeters need repairs, are locked away, etc.) rather than the health worker’s choice?

**Where is the study taking place, how many people does it involve and how are they selected?**

The study will take place in two hospitals where pulse oximeters are used and two hospitals where pulse oximeters are not used, and will involve up to 25 semi-structured interviews with nurses and doctors. Participants will be chosen based on their health profession, and through consultation with investigators running a larger study exploring how to improve quality of care in Kenyan hospitals (SSC 2465: Prof. M English and Prof. G. Irimu).

**What does the study involve for those who are in it?**

The participants will each be interviewed for between 30 minutes and one hour.

**What are the benefits and costs of the study for those involved?**

There are no costs or direct benefits for those involved.

**How will the study benefit society?**

It is important to understand what prevents apparently simple and useful technologies from being used as part of routine care. Only by understanding the barriers that prevent use of simple technologies like pulse oximetry will we be able to design better ways of promoting their use. Improving the use of such technologies can then, we hope, help to improve patient care and reduce child mortality rates.

**When does the study start and finish?**

We aim to start the interviews at the end of October / beginning of November 2016 (dependent on the timing of ethical approval) and we anticipate the interviews will take 3 weeks (but may take longer if saturation has not been reached after 3 weeks).

**1. Title of the Project**

Pulse oximeter use in Kenyan hospitals: a qualitative study of health workers’ perspectives of the barriers to pulse oximeter use

**2. Investigators and Institutional Affiliations**

**Principal investigator**

Abigail Enoch: PhD student at Nuffield Department of Population Health, University of Oxford, UK

**Co-investigators**

Prof Mike English and Jacinta Nzinga: KEMRI – Wellcome Trust Research Programme, Nairobi

Prof Grace Irimu and Dr Jacquie Oliwa: KEMRI – Wellcome Trust Research Programme, Nairobi; Department of Paediatrics and Child Health, University of Nairobi

Prof Gerry McGivern: Warwick Business School, University of Warwick, UK

Prof Sasha Shepperd: Nuffield Department of Population Health, University of Oxford, UK

**3. Abstract**

Pulse oximeters are a low-cost tool that could help reduce child mortality by helping health workers determine the severity and nature of illness. However, data collected as part of an ongoing approved study (SCC 2465) through the Clinical Information Network (CIN) suggest some hospitals do not have access to pulse oximeters and that this technology is often not used in Kenyan hospitals, even when they are available. This research is being undertaken to help explain such findings and will involve up to 25 semi-structured interviews with health workers in four Kenyan hospitals, to examine why pulse oximeters may not be available or why health workers use or do not use them when admitting children. This information will be used to help develop effective strategies to encourage appropriate pulse oximeter use.

**4. Introduction/Background**

In newborns, children and adolescents, hypoxemia is associated with increased risk of death, and is a common complication of bronchiolitis, pneumonia, asthma and other serious conditions (e.g. sepsis) [1-4]. Pulse oximetry is a low-cost intervention that could reduce child mortality, in line with the targets of Sustainable Development Goal 3, by enabling early detection of hypoxemia and improving accurate diagnosis, thereby increasing the chance of prompt, effective treatment.

However, despite the potential to improve health outcomes, pulse oximeters are often not available, particularly in low-income settings [5,6], and even when they are available, they are often not used [7-9].

The Kenyan Basic Paediatric Protocols have, since 2013, recommended pulse oximeter use for helping to assess children and determining appropriate therapy especially amongst those with pneumonia, or other seriously sick children [10]. However, there has not been any national program to provide and distribute pulse oximeters so pulse oximeter introduction in Kenyan hospitals has been led at the hospital level, according to informal observations.

As part of previously approved work (SCC 2465) that is investigating how different forms of support including feedback provided to hospitals can help promote the adoption of recommended practices in the care of children with pneumonia, malaria and dehydration, observational data on the use of pulse oximetry has been collected. It is recommended that all children have an admission pulse oximetry measurement taken. Quantitative analyses of these data from 14 hospitals in Kenya show that pulse oximeter use varies substantially across hospitals: one hospital does not have any pulse oximeters (while 2 others recently obtained at least one pulse oximeter); on average, between September 2013 and February 2016, pulse oximeters were not used in four hospitals, while at the other ten hospitals, pulse oximeters were used on admission for 12-71% of children.

Little research in any setting has examined why pulse oximeters are not used, even when available. The limited evidence suggests health workers’ decisions surrounding whether to use pulse oximeters may vary depending on characteristics of children such as age, respiratory symptoms, diagnosis, and date of admission, and that barriers to pulse oximeter use may include lack of training, lack of guidelines, and the need for repairs [8,11-14].

We plan to interview health workers in Kenyan hospitals to ask them about what barriers they identify as preventing them from using pulse oximeters and to ask them why they may choose to use or not to use pulse oximetry when it is available. This research will therefore consider the perspectives of health workers rather than looking at the procurement systems, national efforts or policy issues. (However, these issues will be recorded if they are raised by those interviewed.) This work is conducted to help explain data demonstrating variable pulse oximeter use and is in the context of a national paediatric guideline promoting pulse oximeter use when admitting seriously sick children, e.g. those with pneumonia. The costs for pulse oximeters have recently fallen dramatically and this makes costs less of a concern while implementing the guideline [15-17].

**5. Justification for the study**

Increasing pulse oximeter use could help to improve diagnosis and target treatment and consequently reduce child mortality rates. However, as with many technologies in health, the adoption of pulse oximetry measurement is only partial, and to improve their use, it is first necessary to understand: i) why hospitals have and have not introduced pulse oximeters, ii) for what reasons health workers cannot access pulse oximeters even when these are made available (e.g. pulse oximeters need repairs or are locked away, etc.) iii) how health workers choose for which children to use pulse oximetry. These aspects will be explored during the interviews and the findings will help explain the observation of variable pulse oximetry use (across hospitals and across children with different characteristics) emerging from other studies (SCC 2465).

The results from this qualitative work will help develop effective strategies to overcome barriers and to promote pulse oximeter use in Kenyan hospitals. Ultimately this could help improve treatment and reduce child mortality rates.

**6. State the Null Hypothesis**

There is no null hypothesis as this is a qualitative interview study.

**7. Objectives**

**a) General Objective**

To explore the perspectives of health workers on why some Kenyan hospitals have adopted pulse oximetry and why health workers with access to pulse oximeters do or do not use them.

**b) Specific Objectives**

- To understand why pulse oximeters have been adopted in some hospitals but not others
- To understand how health workers decide to use or not use pulse oximeters with children at admission
- To understand the barriers that may prevent health workers from being able to choose to use a pulse oximeter with children at admission (e.g. the pulse oximeters are broken)
- To understand how health workers’ decisions about treatment provision are affected by pulse oximeter results

**8. Design and Methodology**

We have used the Consolidated Framework for Implementation Research (CFIR) to help inform our approach, and to develop the interview topic guide questions [18]. The CFIR is a framework that combines parts of a range of other models to demonstrate the factors that may impact implementation of a public health intervention, such as a medical tool, behavioural change, etc. It contains components from 20 sources, including a previous review of 500 sources of potential factors that impact intervention implementation. These factors are grouped into five categories: a) intervention characteristics (which in the context of this research would include, for instance, how effective pulse oximeters are, how easy to use); b) outer setting (e.g. the national guidelines for pulse oximeter use); c) inner setting (e.g. policies of the health facility concerning pulse oximeter use, the funding source for pulse oximeters); d) characteristics of individuals (e.g. the knowledge, perceptions, attitudes and beliefs of the health workers who are recommended to use pulse oximeters, concerning pulse oximeter use and associated provision of care in general); e) process (e.g. whether opinion leaders were engaged when pulse oximeters were first introduced into hospitals, what training was provided in how/when/why to use pulse oximeters) [18]. See Appendix 1 for more information about these categories. The CFIR’s varied, inclusive and multi-faceted nature make it ideal for considering decision-making and barriers surrounding pulse oximeter use in Kenyan hospitals, which are likely to be impacted by an interplay of numerous factors.

**a) Study Site**

Interviews will be conducted at the following four hospitals: {*names* *removed from protocol before publication in PLOS Medicine for ethical reasons*}. All of these hospitals are public hospitals that are involved in a currently running study (SSC 2465) that provides basic data on the proportion of the paediatric patient population in which pulse oximetry is used. These hospitals were selected purposefully, in consultation with investigators running the main study (SSC 2465), to represent varying contexts (e.g. different areas, different patient volumes).

Pulse oximetry has not been adopted at two of the included hospitals; these hospitals will be included to explore the reasons for this (interviews will be conducted with at least two senior health workers such as the paediatrician and nursing officer in charge in each hospital). Two hospitals using pulse oximetry will also be included to explore why pulse oximetry is or is not used when it is available (a minimum of six interviews will be conducted in each hospital; see section on study population).

Hospitals are thus selected to illustrate varying contexts rather than as an effort to gain a representative sample. Overall our aim is to conduct interviews that ensure a wide diversity of views is captured.

**b) Study Populations**

The paediatrician and senior nurse from the paediatric department will be interviewed in hospitals where pulse oximetry is not available. In hospitals where pulse oximetry is available, the paediatrician, the senior nurse, a medical officer, a medical officer intern, a clinical officer intern and a ward nurse will be interviewed. Senior administrative staff (e.g. the medical superintendent or hospital administrator) in all four hospitals may also be interviewed if early findings suggest this will be important (e.g. if interviewees indicate that administrative factors encourage or provide barriers to pulse oximeter adoption/use).

**c) Recruitment and Sampling**

Within hospitals, potential participants will be identified through purposive sampling during the period the researcher visits the hospital; participants will be identified based on health profession and availability for an interview; there will be no exclusion criteria. A paediatrician and senior nurse will be recruited from the paediatric department at each hospital where pulse oximetry is not available; and a paediatrician, senior nurse, medical officer, medical officer intern, clinical officer intern and ward nurse, identified when the investigator visits the hospitals, will be recruited from the paediatric department at each hospital where pulse oximeters have been adopted.

Before they are formally included as study participants, each health worker who is approached will be given an information sheet explaining the purposes of the research and how their data will be stored and used, and will be asked to provide written consent to being interviewed. They will be free to decline to be interviewed, with no negative repercussions, and in this case a suitable replacement will be invited.

**d) Procedures**

Across the four hospitals in the study, we will conduct a minimum of 16 interviews and up to 25 semi-structured interviews overall; the number of interviews will be determined by whether or not new insights are being gained as interviews accumulate; once no new understanding is gained (saturation) we will stop.

The topic guides will be piloted on at least one nurse or doctor in a hospital where pulse oximeters are used and in one where pulse oximeters are not used prior to any of the interviews.

Each interview will take between 30 minutes to an hour. Interviews will be conducted in offices or other appropriate areas, at a time convenient to the interviewee and taking care to ensure confidentiality is maintained. The researcher will aim to minimise any disruption to work by arranging interviews during formal breaks (e.g. lunch breaks) or just before or at the end of working shifts. If necessary, some interviews may be conducted by telephone. (If a telephone interview is conducted the study information sheet will be read to the interviewee and verbal assent taken that is documented by the researcher). Interviews will be recorded using two audio-recorders (to avoid problems should one recorder not work), if the interviewee consents to this. If no consent is provided for recording then the researcher will keep notes of the interview.

Interviews will be guided by efforts to address the following questions (note: these are the conceptual questions we are considering, not those that interviewees will be directly asked; see Appendix 2 for the Topic Guide questions):

*For hospital staff where pulse oximetry has not been adopted*

- Has the introduction of pulse oximetry to this hospital been discussed or attempted (tell me about any of these discussions/efforts)? Why do you think there is no pulse oximetry in use in paediatric care in this hospital?
- Why do you think there is differential access to this technology across hospitals?

*For hospital staff where pulse oximetry has been adopted*

- How was pulse oximetry introduced into your hospital? Was this influenced by any national or county policies or actions or was it local to this hospital?
- Why do you think there is differential access to this technology across hospitals?
- In what situations do health workers in your hospital choose to use or not use pulse oximeters when seeing children admitted to the hospital, and why?
- In what situations do health workers in your hospital want to use pulse oximeters but cannot when seeing children admitted to the hospital, and why?
- How does using pulse oximetry impact the clinical decision-making of health workers in your hospital?
- How were your hospital’s pulse oximeters funded?

See Appendix 2 for a more formal topic guide.

**9. Data Management and Analysis**

Interviews will be transcribed by the lead researcher. The researcher will then code the transcripts using thematic content analysis. This will involve developing low then medium then high level codes, of an increasingly general nature, through an iterative process; the transcripts will be read multiple times at each stage to ensure that the collected data truly support the codes and themes and that no codes and themes are left out. Co-investigators will also read transcripts and check or amend the coding as indicated.

The development of codes will be guided by the Consolidated Framework for Implementation Research. We will be open to other themes emerging from the data and may use other theories in addition to the CFIR, to help inform our analyses, depending on the data collected in the interviews. For instance, if it is found in the interviews that health workers’ decisions are affected by the views of those higher in the hierarchy or by other social or organisational factors, then we may consult theories associated with the sociology of professions or organisations.

This analysis process will be done manually. Two or three quotes will be chosen to exemplify each theme.

The results of the analyses will be explored for their validity by presenting draft findings to hospital personnel from the hospitals from which data are collected, and asking if they think the analyses are accurate and appropriate.

A summary of the final results, analyses and conclusions will also be given to the participants and to the management of the hospitals involved with the research, ensuring no place or person can be identified.

**10. Intellectual Property**

Any Intellectual property rights that arise from the work will be safeguarded according to the KEMRI IPR Policy of 2015 and the Industrial Property Act of 2001, sections 32, 58 and 80. The scientific and intellectual contributions of all persons involved in the research will be appropriately acknowledged in all publications and presentations arising from the work.

**11. Timeframe**

The interviews are expected to take 3 weeks with 2-5 days spent in each hospital, but will take longer if this is needed to arrange and conduct interviews and if saturation has not been reached by the end of three weeks. We aim to conduct the interviews in late October / early November 2016, but this is dependent on the timing of ethical approval. Analysis will then continue for 2-3 months before writing of results. In all, the project is expected to take about 6 months.


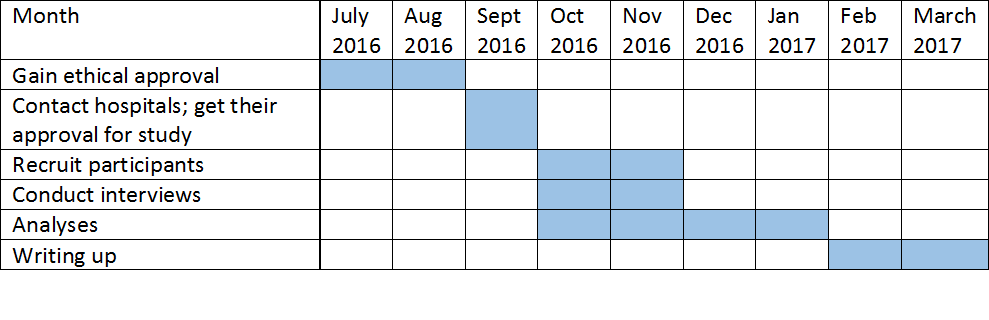


Note: the recruiting of participants and conducting of interviews may take longer than 3 weeks if saturation has not been reached by the end of 3 weeks

**12. Ethical Considerations**

This research is subject to review by SERU, the ethical committee of the University of Oxford’s Medical Sciences Department, and the hospital authorities of the target hospitals. No research will begin until these approvals are obtained.

Taking part in this study and agreeing to be interviewed is entirely voluntary and there will be no penalty for declining to participate. Participants can choose not to answer any of the questions for any reason and can end the interview at any point for any reason and with no penalty. Participants can also request for their interviews to be deleted, with no record retained.

**a) Confidentiality**

Only individuals directly involved with this research will have access to participants’ information. We will not share any information about participants beyond a few individuals directly involved in the study.

Participants will be given ID codes; only this code, no names or other identifiers, will be written for identification on the transcripts; the ID code legend linking names to ID codes will be kept in a secure location separate from the transcripts.

Any quotes used in the analysis will not have names or other identifiers that could indicate the identity of the speaker; quotes with very specific information that could identify the speaker will not be used.

Data will be securely stored in accordance with the policies of KWTRP and Oxford’s Nuffield Department of Population Health; transcripts and recordings of interviews will be stored on an encrypted password-protected laptop and an encrypted USB drive; and only named investigators will have access to the data.

Recordings of the interviews and the legend linking names with ID codes will be deleted after the end of the study. Anonymized transcripts will be placed in the secure archives of KWTRP and Oxford University for three years after the end of the study. Any further use of these data will only be allowed with appropriate ethical approval. After this period these transcripts will be destroyed.

**b) Informed consent process**

Before each interview, participants will be given an information sheet explaining: the purposes of the research; that their participation is completely voluntary and that there will be no negative repercussions from participating, declining to participate or giving a particular response; and how their data will be confidentially stored and used. (See Appendix 3 for this information sheet). Participants can keep this information sheet for future reference. This information will also be discussed with the participant, and the researcher will ask if the participant has any questions. The participant will then be asked to sign a consent form in duplicate to agree to being interviewed and for their responses to be used in this research. (See Appendix 4 for this consent form). The participant will be given one of these signed consent forms to keep and the researcher will keep the other signed consent form. Interviews will be recorded on two electronic recorders (to ensure no responses are lost or misremembered), if the participant consents to having the interview recorded. If no consent is provided for recording then the researcher will keep notes of the interview.

**c) Benefits and costs**

There are no individual benefits to taking part and no reimbursement or payment of any kind will be given. The benefits from this research will be to provide information on pulse oximeter use and barriers to use. This evidence will be made available to hospitals in Kenya and to the partners in this research that include the University of Nairobi and the Kenya Paediatric Association. Two of the co-investigators of this research (ME and GI) collaborate closely with these partners and the Ministry of Health to help develop national paediatric guidelines and implementation strategies to promote the use of these guidelines, so this research will hopefully inform the development of effective strategies to support appropriate pulse oximeter use.

The cost from this research for the interview participants is the time taken to do the interview; we will make every effort to minimise disruption to the work of staff. There will be no other costs from this research for the interview participants or hospitals.

**d) Feedback of findings**

The results of the analyses will be triangulated by presenting draft findings to hospital personnel from places where data are collected, and asking if they think the analyses are accurate and appropriate.

A summary of the final results, analyses and conclusions will also be given to the participants and to the management of the hospitals involved with the research, ensuring no place or person can be identified.

We will also look for suitable opportunities to feedback the findings of this research to the wider paediatric community e.g. through the annual Kenya Paediatric Association conference, through presentation at the University of Nairobi and through meetings with the Ministry of Health.

**e) Data sharing from this study**

The summary and anonymized results of the analyses will be shared with the participants and the included hospitals. This information will also be included in the thesis of the primary investigator and will be shared with the wider research community through publications and presentations.

Data from the interviews will only be available to the named research investigators to ensure confidentiality of participants. Requests for access to primary data from these interviews by people other than the investigators will be submitted to the KWTRP data governance committee as a first step, who will advise on the need for additional ethical review by the KEMRI Research Ethics Committee.

**f) Community engagement**

The study has been discussed with the paediatricians working on the larger study on improving quality of care in Kenyan hospitals, and also with those in the Ministry of Health who are responsible for developing child health policy at national levels. Lessons learned will be communicated to them and the wider Kenyan Paediatric Association community through its annual meeting.

Local doctors, nurses and hospital management (the hospital management team) will have the research explained to them before any data collection. The findings of this research will be provided as part of the process of checking their validity.

**g) Work on animals**

This research will not involve animals.

**13. Expected Application of the Results**

These results can be used to understand why adoption of pulse oximetry varies between hospitals and may not be consistent across patients within a hospital. Such understanding can help in developing effective strategies to remove barriers to pulse oximeter use and promote pulse oximetry, which in turn can help to improve diagnosis and treatment and potentially contribute to reduced child mortality rates. The long-term work that the KWTRP team has done with professionals and the Ministry of Health to create national clinical guidelines for paediatric care will support use of the findings to improve clinical practices.

**14. Study limitations**

The study could be strengthened by employing additional qualitative methodologies such as focus groups or observations of pulse oximetry use in practice (which would enable further triangulation), or by prospectively exploring reasons for use/not-use; also investigating pulse oximeter use, barriers and promotion efforts at the logistical, administrative and political levels would broaden understanding of the topic. However, these approaches will not be feasible due to time and funding limitations. Nevertheless we feel that the proposed data will still be very useful for the hospitals to gain a better understanding of how they can encourage pulse oximeter use, particularly as these results will be complementing the quantitative analyses we are conducting with data on admissions of children to Kenyan hospitals.

All qualitative research has the limitation that there is a risk that participants will tell the interviewer what they think the interviewer wants to hear. We will aim to minimize this bias by a) ensuring participants are as comfortable as possible (e.g. starting the interview with questions about what they enjoy about their job), explaining that there are no right answers as we are interested in their opinions and experiences, and reassuring them about the confidentiality of the interviews; b) triangulating information across interviews, as well as with informal observations; c) being aware of the potential for this bias and accordingly being reflexive in the analyses. In our interpretation of the results we will therefore be trying to draw insight from across interviews to uncover likely mechanisms, rather than claiming absolute truths or the magnitude of effects.

**15. References**

1. World Health Organization. Manual on use of oxygen therapy in children. 2014.

2. Lozano JM. Epidemiology of hypoxaemia in children with acute lower respiratory infection [Oxygen Therapy in Children]. The international journal of tuberculosis and lung disease 2001;5(6):496-504.

3. Djelantik IG, Gessner BD, Sutanto A, Steinhoff M, Linehan M, Moulton LH, et al. Case fatality proportions and predictive factors for mortality among children hospitalized with severe pneumonia in a rural developing country setting. Journal of tropical pediatrics 2003;49(6):327-332.

4. Orimadegun A, Ogunbosi B, Orimadegun B. Hypoxemia predicts death from severe falciparum malaria among children under 5 years of age in Nigeria: the need for pulse oximetry in case management. African health sciences 2014;14(2):397-407.

5. Desalu OO, Onyedum CC, Iseh KR, Salawu FK, Salami AK. Asthma in Nigeria: Are the facilities and resources available to support internationally endorsed standards of care? Health Policy 2011;99: 250-254.

6. English M, Gathara D, Mwinga S, Ayieko P, Opondo C, Aluvaala J, et al. Adoption of recommended practices and basic technologies in a low-income setting. Archives of Disease in Children 2014;99:452-456.

7. McCollum ED, Bjornstad E, Preidis GA, Hosseinipour MC, Lufesi N. Multicenter study of hypoxemia prevalence and quality of oxygen treatment for hospitalized Malawian children. Transactions of the Royal Society of Tropical Medicine and Hygiene 2013;107(5):285-92.

8. Ginsburg AS, Gerth-Guyette E, Mollis B, Gardner M, Chham S. Oxygen and pulse oximetry in childhood pneumonia: surveys of clinicians and student clinicians in Cambodia. Tropical Medicine and International Health 2014;19(5):537-544.

9. Ginsburg AS, Van Cleve WC, Thompson MIW, English M. Oxygen and Pulse Oximetry in Childhood Pneumonia: A Survey of Healthcare Providers in Resource-limited Settings. Journal of tropical pediatrics 2012;58(5):389-393.

10. Kenyan Ministry of Health. Basic Paediatric Protocols for ages up to 5 years. 2013.

11.Pham JC, Kelen GD, Pronovost PJ. National study on the quality of emergency department care in the treatment of acute myocardial infarction and pneumonia. Academic emergency medicine : official journal of the Society for Academic Emergency Medicine 2007;14(10):856-863.

12. Jones M. The Oxygen Tissue Saturation (SPO2) Audit. Paediatric Research 2010;68:239.

13. Berkley JA, Brent A, Mwangi I, English M, Maitland K, Marsh K, et al. Mortality among Kenyan children admitted to a rural district hospital on weekends as compared with weekdays. Pediatrics 2004;114(6):1737-1738.

14. English M, Ntoburi S, Wagai J, Mbindyo P, Opiyo N, Ayieko P, et al. An intervention to improve paediatric and newborn care in Kenyan district hospitals: understanding the context. Implementation Science 2009;4:42.

15. Kenyan Ministry of Health. Basic Paediatric Protocols for ages up to 5 years. 2016;4th edition.

16. Lifebox. Value of a Lifebox. 2015; Available from: <http://www.lifebox.org/safe-surgery/value-of-a-lifebox/>.

17. Peterson CL, Chen TP, Ansermino M, Dumont GA. Design and evaluation of a low-cost smartphone pulse oximeter. Sensors 2013;13(12):16882-16893.

18. Damschroder LJ, Aron DC, Keith RE, Kirsh SR, Alexander JA, Lowery JC. Fostering implementation of health services research findings into practice: a consolidated framework for advancing implementation science. Implementation Science 2009;4:50.

**16. Role of Investigators and Advisors**

**Investigators**

AE will be conducting the interviews.

**Co-Investigators**

ME, JN, GI and JO have built up considerable experience in health systems research in Kenya and provide links to the University of Nairobi, the Kenya Paediatric Association and the Ministry of Health. They have undertaken or supervised previous research aiming to understand barriers to the provision of good quality care. They will read interview transcripts and check thematic coding and contribute to analysis and writing up.

GM and SS have experience with qualitative research and social and organisational theories as well as theories of implementation and will therefore advise on interview technique and the transcript coding and analysis, and will contribute to writing up.

**17. Appendices**

**Appendix 1: Consolidated Framework for Implementation Research table**

Adapted from the Center for Clinical Management Research’s description of the Consolidated Framework for Implementation Research, which can be found here: <http://www.cfirguide.org/index.html>

| **Categories of constructs** | **Explanation of category** | **Constructs** | **Example(s) in the context of this research** |
| --- | --- | --- | --- |
| Intervention characteristics | The components and features of the intervention | Intervention source; evidence strength & quality; relative advantage; adaptability; trialability; complexity; design quality and packaging; cost | Whether pulse oximeters are accurate; whether they are easy to use; how they are charged/what kind of batteries are needed; what they cost |
| Outer setting | The economic, political and social context which the organization resides within | Patient needs & resources; cosmopolitanism; peer pressure; external policy & incentives | The national guidelines for paediatric care |
| Inner setting | The structural, political and cultural context within which the intervention proceeds | Structural characteristics; networks & communications; culture; implementation climate (tension for change, compatibility, relative priority, organizational incentives & rewards, goals & feedback, learning climate); readiness for implementation (leadership engagement, available resources, access to knowledge and information) | The regulations and policies of the hospitals; the funding sources for pulse oximeters |
| Characteristics of individuals | The knowledge, perceptions, attitudes and beliefs of those involved with the intervention; how these affect the decisions and actions of others; and the interplay between individuals and the organisation | Knowledge & beliefs about the intervention; self-efficacy; individual state of change; individual identification with organization; other personal attributes | Health workers’ knowledge of how to use pulse oximeters appropriately; whether they think pulse oximeters are important, accurate and easy to use (particularly compared to other methods); when they think it is useful to use pulse oximeters; how decisions of use/non-use by one / a group of health workers affects the decisions of other health workers |
| Process | How the intervention is introduced and carried out | Planning; engaging (opinion leaders, formally appointed internal implementation leaders, champions, external change agents); executing; reflecting & evaluating | What training was provided on how to use pulse oximeters appropriately; what efforts there have been to promote pulse oximeter use |

**Appendix 2: topic guide questions**

The topic guides will be piloted on at least one nurse or doctor in a hospital where pulse oximeters are used and in one where pulse oximeters are not used prior to any of the interviews.

***For hospital staff where pulse oximetry is not used***

- What bedside diagnostic tools do you use to help figure out the diagnosis and disease severity of children admitted to your hospital?

-How do you choose which tool to use?

- What are the steps that you take if you think that an admitted child may have pneumonia?
- How do you decide if you think that a child has low oxygen levels in the blood?
- How do you decide when to give oxygen to a child?
- What do you think of pulse oximeters?

-How useful do you think they are?

-What do you think they are most useful for?

-How easy to use do you think they are?

- In what situations do you think you would choose to use a pulse oximeter if one was available?
- In what situations do you think it would not be useful or necessary to use a pulse oximeter even if available?
- Can you please tell me about oxygen availability in your hospital?
- Can you please tell me about any discussions or efforts there have been to try to introduce or encourage pulse oximetry at this hospital?
- Why do you think there is differential access to pulse oximeters across hospitals?

***For hospital staff where pulse oximetry is used***

- What bedside diagnostic tools do you use to help figure out the diagnosis and disease severity of children admitted to your hospital?

-How do you choose which tool to use?

- What are the steps that you take if you think that an admitted child may have pneumonia?
- How do you decide if you think that a child has low oxygen levels in the blood?
- How do you decide when to give oxygen to a child?
- What do you think of pulse oximeters?

-How useful do you think they are?

-What do you think they are most useful for?

-How easy to use do you think they are?

- In what situations do you generally choose to use a pulse oximeter?
- In what situations do you think it is not necessary or useful to use a pulse oximeter?
- Can you please tell me about oxygen availability at your hospital?

-What problems, if any, do you have with accessing oxygen?

-How does oxygen availability affect your decision to use pulse oximeters, if at all?

- In what situations, if any, do you find that you want to use a pulse oximeter but are not able to?

-How often does this occur?

-How has this issue changed over time, if at all?

-What do you think could help to solve this problem?

-Could you please tell me about any other equipment that you have similar issues with?

-How do you think other doctors or nurses feel about this problem?

- So when you do use a pulse oximeter, in what ways, if any, do you think the results affect your opinions or decisions?

-How about your decisions about diagnosis?

-How about your decisions about treatment?

- When did you first hear about pulse oximetry?

-What form of training, if any, was provided then?

-What did you think of pulse oximetry after this introduction?

- How was pulse oximetry introduced in your hospital?

-Was this influenced by any national or county policies or actions or was it local to this hospital?

-What form of training, if any, was provided then?

-What did you think of pulse oximetry after this introduction?

- How often do you think other doctors or nurses in your hospital use pulse oximeters?
- For what reasons do you think other doctors in your hospital choose to use or not use pulse oximeters?
- Why do you think there is differential access to pulse oximeters across hospitals?
- How are the pulse oximeters that are available at your hospital funded?

**Appendix 3: information sheet to be given to participants during the consent process**

**KEMRI Wellcome Trust Research Programme: Participant Information Sheet**

**Study title: Pulse oximeter use in Kenyan hospitals: a qualitative study of health workers’ perspectives of the barriers to pulse oximeter use**

| Institution | Investigators |
| --- | --- |
| Nuffield Department of Population Health, University of Oxford | *Abigail Enoch, Prof Sasha Shepperd* |
| Nuffield Department of Medicine, University of Oxford; KEMRI – Wellcome Trust Research Programme, Nairobi, Kenya | *Prof Mike English, Jacinta Nzinga* |
| Department of Paediatrics and Child Health, University of Nairobi | *Prof Grace Irimu, Dr. Jacquie Oliwa* |
| Warwick Business School, University of Warwick | *Prof Gerry McGivern* |

**Who is carrying out this study and what is this study about?**

This study is being conducted as part of a collaborative project between the University of Oxford, the Kenya Medical Research Institute (KEMRI) Wellcome Trust Research Programme, the Kenya Paediatric Association and the Ministry of Health. KEMRI is a government organization that carries out medical research to find better ways of preventing and treating illness in the future for everybody’s benefit. Sometimes research involves only asking questions of patients, their parents, community members or health providers about what they know, feel or do.

In this research, we want to learn more about how health workers determine the diagnosis and disease severity of children admitted to hospitals, including health workers’ perspectives of appropriate use of and barriers to pulse oximetry; we want to listen to your opinions, and ask you to explain your decision-making process and experiences related to this issue. We would like to hold individual interviews with up to 25 health workers who work with children in Kenyan hospitals.

The study is being done at four hospitals involved in a larger study on improving quality of care in Kenyan hospitals.

**Why do you want to talk to me and what does it involve?**

Given your experience as a health worker working with children in a hospital in Kenya, you can contribute much to our understanding and knowledge of how children are assessed at admission to hospital.

- I would like to ask you a number of questions about how you try to figure out the diagnosis and disease severity of children when they are admitted to the hospital, what medical tools or equipment you use to do this (e.g. stethoscope, temperature measurements, pulse oximetry, etc.), and how you choose which actions to take.
- If you do not want to answer any of the questions you may say so and I will move on to the next question.
- The interview will take between 30 minutes and one hour. It will take place at a time and place that is convenient for you.
- No-one else but I will be present unless you would like someone else there.
- If you agree, the interview will be recorded using two audio-recorders to assist later in fully writing up the information. No-one will be identified by name in the recording.

**Are there any disadvantages or advantages to me of taking part?**

The interview will take between 30 minutes to one hour of your time. You are free to stop the discussion or leave the study at any point if you feel this is necessary. You are also free to not answer any question you feel uncomfortable with.

There are no individual benefits to taking part and no reimbursement or payment of any kind will be given. However in talking to us, you will contribute to knowledge of assessment of child admissions that may help other people in Kenya and elsewhere in the future, for example through developing new health policies or informing the design of interventions aimed at assisting health workers in improving care for children.

**Who will have access to the information I give?**

- Only individuals directly involved with this research will have access to your information. We will not share any information about you or about any other research participant beyond a few individuals directly involved in the study.
- All audio-recordings and interview transcripts will be stored securely on password protected computers only accessible to concerned research staff.
- Audio-recordings will be deleted after the end of the study. Transcripts will be placed in the KEMRI and University of Oxford secure archives for three years and then will be destroyed.
- Every participant will be assigned a unique identifier to preserve anonymity.
- The knowledge gained from this research will be shared in summary form, without revealing individuals’ identities, with participants and the hospitals where you work; this summary information may also be presented at seminars or conferences and be submitted to a journal for publication. In all cases, we will only share information in ways that do not reveal individual participants’ identities. For example, we will remove information that could identify people, such as their names and where they work, and replace this information with number codes.
- Any future research using information from this study must first be approved by the national expert committee to make sure that the interests of participants and their communities are protected.

**Who has allowed this research to take place?**

All research at KEMRI has to be approved before it begins by several national and international committees who look carefully at planned work. They must agree that the research is important, relevant to Kenya and follows nationally and internationally agreed research guidelines. This includes ensuring that all participants’ safety and rights are respected.

This research will not take place until ethical approval has been obtained from the ethical committee of the Kenya Medical Research Institute, the ethical committee of the University of Oxford’s Medical Sciences Department, and the hospital authorities of your hospital.

**What will happen if I refuse to participate?**

All participation in research is voluntary. You are free to decide if you want to take part or not. If you do agree you can change your mind at any time without any consequences.

**What if I have any questions?**

You are free to ask me any question about this research. If you have any further questions about the study, you are free to contact the research team using the contacts below:

Prof Mike English and Dr. Jacquie Oliwa, KEMRI Wellcome Trust Research Programme, P.O. Box 43640-00100, Nairobi. Telephone: 730 162000 or 041 7522063

**If you want to ask someone independent anything about this research please contact:**

Community Liaison Manager, KEMRI Wellcome Trust Research Programme, P.O. Box 43640-00100, Nairobi. Telephone: 730 162000 or 041 7522063

***And***

The Secretary - KEMRI/Scientific and Ethics Review Unit, P. O. BOX 54840-00200, Nairobi, Tel number: 020 272 2541 Mobile: 0722 205 901 or 0733 400 003 or 7171 719477

**Appendix 4: consent form**

**KEMRI-Wellcome Trust Research Programme consent form for Pulse oximeter use in Kenyan hospitals: a qualitative study of health workers’ perspectives of the barriers to pulse oximeter use**

I have had the study explained to me. I have understood all that has been read/explained and had my questions answered satisfactorily.

**⬜ Yes** *please tick* **I agree to be interviewed**

**⬜ Yes** *please tick* **I agree for the interview to be audio-recorded**

I understand that I can change my mind at any stage and it will not affect me or my work in any way.

**Signature:** __________________________________ **Date:** _________________

**Participant name:** _____________________________ **Time:** _________________

***(please print name)***

---------------------------------------------------------------------------------------------------------------------------

I certify that I have followed the study SOP to obtain consent from the participant. S/he apparently understood the nature and the purpose of the study and consents to participation in the study. S/he has been given opportunity to ask questions which have been answered satisfactorily.

**Designee/Investigator’s signature:** ______________________ **Date:** _____________

**Designee/Investigator’s name:** __________________________ **Time:** _____________

***(please print name)***

# THE PARTICIPANT SHOULD NOW BE GIVEN A SIGNED COPY TO KEEP

*……………………………………………………………………………………………………………*
