# Supplementary figures and images for: Variability in the use of pulse oximeters with children in Kenyan hospitals: A mixed-methods analysis
Source: PLoS Med. 2019 Dec 31;16(12):e1002987. doi: 10.1371/journal.pmed.1002987 (PMC6938307; doi:10.1371/journal.pmed.1002987)

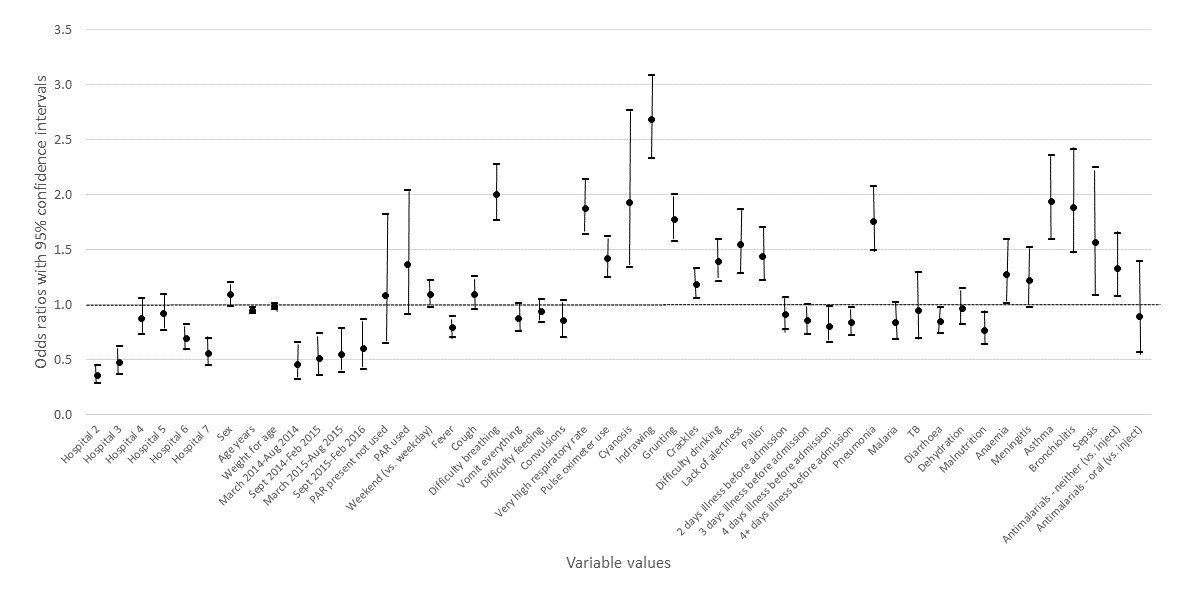

Supplement: S1 Fig — OR, odds ratio. (TIF) [file pmed.1002987.s010.tif]
