# Supplementary material for: Variability in the use of pulse oximeters with children in Kenyan hospitals: A mixed-methods analysis
Source: PLoS Med. 2019 Dec 31;16(12):e1002987. doi: 10.1371/journal.pmed.1002987 (PMC6938307; doi:10.1371/journal.pmed.1002987)
Supplement: Attachment — Submitted filename: Responses to reviewers, final.docx [file pmed.1002987.s012.docx]

Responses to reviewers’ comments

We would like to thank the reviewers very much for the thoughtful and helpful comments and suggestions.

We have modified the manuscript to take these into account, and have listed below our responses to the comments and suggestions, the edits we have made, and the rationales for the edits.

Please see the document with changes shown in altered colours to see the changes that have been made to the manuscript. Changes shown in green, blue or pink refer to changes made in response to specific reviewer’s comments (see below). Changes in orange are changes made in response to multiple reviewers, or more general rephrasing or re-ordering for clarity purposes.

Reviewer #1: [changes in response to these comments are shown in green in the paper]

| Reviewer’s comments | Our response |
| --- | --- |
| Abstract:  Methods - framework guided your design and then you conducted quan and qual data collection to answer specific outcomes - please describe this more clearly.  Findings - inconsistent with findings and conclusions (conclusion seems the clearest so perhaps use this).  Conclusions - first sentence is not a conclusion | We have revised the wording to describe how we used the framework of the Integrative Model of Behavioral Prediction: “*We adapted the Integrative Model of Behavioral Prediction to link the results from the multi-variable regression analyses to the qualitative findings*.”  We confirm that the abstract findings are consistent with the findings and conclusion.  We have revised the abstract conclusion to be consistent with the findings reported in the results section. |
| Page 4 L72 infectious cause of disease | Change made as suggested |
| P4 L82 - include other references here - such as H Graham and King et al | The Graham and King papers were already referenced elsewhere in the paper. We have added references to them in the specified place as well. |
| P5 L112 - add more about the settings, hospitals included and rationale for this | We have added a flow diagram with details of the hospitals included in the quantitative and qualitative analyses (Fig 2).  We have added further details of these hospitals in the S1 Table supplementary document and have referenced this in the paper’s text.  We have also moved information from the data management and analysis section to the results section to describe the setting of the seven hospitals that met the eligibility criteria of pulse oximeters being used with at least 20% of children: “*We analysed data that were collected from seven of the CIN hospitals that had used pulse oximeters with at least 20% of children admitted to the hospital. The hospitals were located in five counties, and between 20-50% of their catchment population were living in extreme poverty*.”  We have added the following to the qualitative methods section to help clarify which hospitals were used in the analyses: “*We collected qualitative data from all 14 CIN hospitals*.” |
| P6 L125 onwards - need to much more clearly explain the methods used and specifically describe the variables used for quan analysis (perhaps list in appendix) and also the qual methods - you mention "direct (non-participant) observations" - what are these and what data did you collect and how did you analysis it and where is it presented and discussed? Also you mention "semi-structured conversations" but you did have topic guides so they are more than conversations? | The variables used for the quantitative analysis are now listed in the S5 Table supplementary document. Also, we have added a more detailed explanation of the methods used to develop the regression model in the S3 text supplementary document.  Added to the methods: “*our initial observations helped refine the topic guide.*”  Added to the methods, when discussing the observations: “*to obtain an understanding of the paediatric admission process, and to inform the content of the topic guide”*  We edited the section in the methods that describes the types of interviews to the following: “*This was followed by 16 semi-structured audio-recorded and transcribed interviews with HCWs (paediatricians, Medical Officers/Interns, Clinical Officers/Interns, and nurses) from four hospitals; shorter interviews with two Procurement Officials and one Medical Superintendent from two of these four hospitals and, at a network meeting, with 11 HCWs from 10 other hospitals. Both types of interviews covered similar topics. The shorter interviews were less formal, lasted up to 15 minutes vs. 20-35, and were recorded through field notes rather than audio-recordings. Both sets of interviews contributed to the findings; quotes were taken from the longer audio-recorded interviews. These will henceforth all be referred to as interviews*.” |
| P10 - Super interesting data but perhaps you can find a better way to present this | We have retained the graph in Figure 1 (now numbered Fig 3), and added text prior to the graph to better explain the findings: “*Overall there was some evidence of an increased use followed by a decrease, and then a subsequent further increase*”. The other two peer reviewers did not raise concern about the presentation of the data in this figure. |
| P11 - Study population - great data here - would be nice to have a flow to show this data and the outcomes etc. - 45% pneumonia is extremely high and should have been discussed more - especially since you only saw 10% hypoxemia? | We have added a flow diagram at the start of the results section (Fig 2), which shows the characteristics of the hospitals and the characteristics of the quantitative and qualitative samples. |
| P11 L216 onwards I am not sure this is the clearest way to present results - I would keep quant and qual separate and bring them together in the discussion - also list OR in descending order and again look at diagram and perhaps explain why you have selected these variables from all 45? | We have retained the current reporting structure, as this was not identified as a problem by the other two peer reviewers. We report the quantitative results followed by the relevant qualitative findings, and have revised the wording at the start of the sections that report the qualitative findings to make the transition between quantitative and qualitative findings clearer, and to make clearer why we feel that linking the two is a helpful strategy for presenting the results.  We have now listed odds ratios in descending order throughout the paper.  We have added on how the regression models were developed (and therefore why we selected these variables) to the S3 Text supplementary document. |
| P14 Use more headings and not sure how you are referencing qualitative data - I would use longer quotes as it is hard to understand the data as presented | We have added the text “*qualitative findings indicate*” and other similar phrases at the start of sections that report the qualitative findings, to make reporting clearer.  We have considered the inclusion of longer quotes but decided to retain the current selection of quotes as this allows us to provide quotes from more than one participant to illustrate a finding, which indicates the wider generalisability of the qualitative findings. We are balancing the parsimony of presentation and richness of the qualitative data while being conscious of ensuring the paper is not overly long. We have therefore listed the key parts of the interview transcripts to illustrate the points we want to make. The other two peer reviewers did not raise this as a problem.  We have made the referencing of the qualitative data clearer by writing out whether it was a paediatrician, Medical Officer / Medical Officer Intern, Clinical Officer / Clinical Officer Intern or nurse who spoke each quote. |
| P19 L373 how the individual uses the pulse oximeter , recognising… | We have rephrased this paragraph to make it clearer. |
| P20 Great diagram - I would show the original up front in the methods and then show the results one here | We have added the original model to the methods section (Fig 1). |
| P21 Discussion - again please check this relates to the data being presented - e.g. L385 the main barriers - is this presented in the data earlier? L394 'leadership role played by senior doctors' again would be good to present this in the results first and have some quotes | The main findings we highlight in the discussion are reported in the results section. For example: in the peer reviewed version of the manuscript an inadequate supply is reported on lines 296 to 315 (at times this is referred to as inadequate availability); broken pulse oximeters are reported on lines 286 to 291, 309 to 315, 321 to 329; and insufficient training is reported on lines 344 to 365. Leadership is reported on lines 238 to 239.  In addition we have added the following to the results section: “*Thus some HCWs explained that their consultants “really insist on the importance”[11;Clinical Officer] of using pulse oximeters, and “every day they ask, how much was the pulse oximetry.”[9;Medical Officer] The result is that “It’s like a chain, yeah; the consultant ask me, and me I ask the intern, how come you didn’t do this. So it becomes like a norm or a culture, like everyone has to do SpO2 for every baby.”[9;Medical Officer] “It happens from the top.”[8;Clinical Officer]*” |
| P22 L414 Does the data not also show something about adherence to guidelines? Would be interesting to discuss this and how it seems they are following iMCI and improving over time? Lots of data showing this is not always the case | In terms of adherence to guidelines:  -We discuss that *“many interviewees independently identified the 90% threshold that is recommended in the Kenyan Protocols as the one they use in daily practice”*  -We state that “*The children most likely to receive oxygen as a result of a low pulse oximeter reading is largely consistent with the oxygen provision guidelines of the WHO’s 2016 Oxygen Therapy for Children manual and the Kenyan Basic Paediatric Protocols*.”  -We state that: “*Guidance was also found to be influential; HCWs frequently mentioned the Kenyan Basic Paediatric Protocols, appeared to trust them, and spoke about an increased awareness of the Protocols amongst the staff.*”  -We discuss that pulse oximeter use on average increased over time in the hospitals and that one of the likely influential factors was the increase in adherence to the Kenyan Basic Paediatric Protocols guidance: “*The dissemination and use of the Kenyan Basic Paediatric Protocols also likely supported the increasing use of pulse oximeters* [23]. |
| P25 Strengths and weaknesses - L496 is this not a limitation as presented now? and the same in P26 L511? | L496: we identified our adaptation of the IMBP model as a strength not a limitation. We however added the caveat that we could have used other models or frameworks.  L511: We added “*One potential limitation of this analysis is that*” at the start of this section to clarify what potential limitation we are addressing in the following sentences. |
| P26/27 Check conclusions against data and align them | We have checked the conclusions against the results, added a number of edits to improve the flow of text and confirm the conclusions align with the reporting of data. |

Reviewer #2 (Statistical Review): [changes in response to these comments are shown in blue in the paper]

| Reviewer’s comments | Our response |
| --- | --- |
| In the abstract, we are told that a PO was more likely to be used "…in certain hospitals, at later time periods, when a Paediatric Admissions Record was used, and if they had a very high respiratory rate (OR: 1.27, 95% CI: 1.13, 1.43)…" It is not clear why ORs for the first 3 associations are not reported, but they are from then on. | The hospital, time period and Paediatric Admissions Record variables were categorical variables where each value for the variable was tested against a baseline (i.e. each time period was tested against the first time period; ‘PAR used’ and ‘PAR present not used’ were tested against ‘no PAR available’; and all hospitals were tested against Hospital 1). This was because we wanted to ascertain if these variables were influential, rather than understanding the more detailed question of variations across each time period, hospital, etc. (which would have necessitated a separate set of analyses). We have now added the odds ratio of PAR used compared with PAR not available to the results. However we do not think it would be appropriate to report the odds ratios for hospital or time period because doing so without explaining the above would be confusing and potentially misleading. However, we have re-ordered the phrasing of this sentence in the abstract so that it is clearer. We have also added a table to the S5 Table supplementary document which includes all of the odds ratios, including those of the hospitals, time periods and Paediatric Admissions record, and have added a reference to this in the results section. |
| The next paragraph of the abstract then starts to talk about the main barriers to PO use, but it is not clear at that point where these results are coming from. Whilst it becomes clearer after reading more of the paper that these are qualitative findings, at the point of reading the abstract, this was not so obvious. | The phrase “*The interviews indicated that*…” has been added before the sentence about the barriers to pulse oximeter use to clarify where these results are from. |
| In the methods, lines 157-158, it states that missing data patterns were explored and the data could be treated as MAR. I often see statements like this, but have yet to find anyone who can explain how this judgement can be made. What was it in the pattern of missing data that was observed that led to this conclusion? Also, the discussion (around line 515) mentions sensitivity analyses that are not described in the methods section, which purportedly tested the MAR assumption. It is not clear what these sensitivity analyses were. | Further details on these analyses have been added to the text and to the S2 Text supplementary document, which we have added a reference to in the identified sections. |
| Multiple imputation for missing data is good to see, but I thought the "M" in MICE stood for "Multiple", not "Multivariate". | According to the Van Buuren & Groothuis-Oudshoorn’s resource which we reference in the paper (van Buuren S, Groothuis-Oudshoorn K. mice: Multivariate Imputation by Chained Equations in R. Journal of Statistical Software 2011;45(3)) and which we used to inform this approach, the “M” stands for “Multivariate”. |
| The last paragraph of the (quantitative) methods section mentions checking the robustness of the results using a bootstrapping method. I can imagine the type of analysis this might entail, but again, there is not enough detail. I wonder whether it would be worth including some more information about these methods (and others noted above) and some results, perhaps in a supplement? | We have created supplementary documents with further details on how the regression models were developed, including the bootstrapping method used (S3 Text), how we determined whether the data could be considered Missing At Random (S2 Text), the descriptive statistics of the children (S4 Table), and the odds ratios and confidence intervals produced from the regression investigating with which children pulse oximeters are used and the regression examining factors affecting oxygen provision (S5 Table and S6 Table respectively). References to these supplementary documents have been added to the appropriate sections. |
| Figure 2 looks quite nice, but is not the best way to show these results. A table would be better, as the magnitude of the odds ratio may not give an accurate impression of the importance of each predictor. | We have added a table showing all of the odds ratios to the supplementary document S5 Table and have referenced this in the results section. |
| Also, there is no table showing the characteristics of the population in relation to all of the factors considered, and summarising the rate of PO use within subgroups - it is important to show the actual data (including missing data levels), and to report the univariate associations with PO use. Showing all of the potential predictors is important so that we can see which predictors made it into the final model, and which did not. | We have added to the supplementary document S4 Table a table showing the characteristics of the population in relation to all of the factors considered, with the rate of pulse oximeter use in each subgroup and the missing data levels. A reference to this table has been added to the appropriate section. |
| The same goes for predictors of supplemental oxygen use; though here we are not even given a figure to summarise the final model. | We have added a table to the supplementary document S6 Table which shows the odds ratios and confidence intervals for the predictors of oxygen provision, and have added a corresponding graph to the supplementary document S7 Figure. We have also added the following sentence to the methods to make it clearer that two models were created to investigate the two questions: “*A model was created to examine factors associated with pulse oximeter use and another, using a similar set of variables, for factors associated with oxygen provision*”. |
| One result mentioned in the paper was that those with a PO reading below 90% were more likely to receive oxygen, with the conclusion being that HCWs were influenced by the recommended 90% threshold. This seems speculative - there could simply be a trend with more oxygen use with lower PO readings (i.e. there may be nothing in particular about the 90% threshold), but this could be investigated to see if the rate of oxygen use changes sharply around the 90% threshold. | We have removed the conclusion that the fact that those with a PO reading below 90% were more likely to receive oxygen indicated that HCWs were influenced by the recommended 90% threshold. Instead this section in the results is as follows: “*The odds of a child being prescribed oxygen was more than twice as high if they had a pulse oximeter reading below 90% than if they had a pulse oximeter reading of 90% or more (OR: 3.29, 95% CI: 2.82, 3.84). Complementing these quantitative results, many interviewees independently identified the 90% threshold that is recommended in the Kenyan Protocols as the one they use in daily practice*”.  Then in the discussion we continue the discussion as follows: “*Interviews indicated that there is an inconsistent use of the 90% threshold, which mirrors the debate continuing in the literature on whether 90% is the threshold that should be used.* [...] *Further research is required on the impact of using the 90% threshold or alternative thresholds on HCW decision-making, health outcomes and resource utilisation*” |

Reviewer #3: [changes in response to these comments are shown in pink in the paper]

| Reviewer’s comments | Our response |
| --- | --- |
| It would be good to tighten the description of the qualitative data sources. It seems there is quant data from 7 hospitals - but qual data from at least 14 hospitals (as described in the abstract)but less clear in the results section itself) plus observation in some. Clearly qual data came form hospitals from which quant data was also collected but it difficult to get a clear pattern of the data sources. Could this be represented in a tabular or other format for clarity? | A figure has been added to the main manuscript (Fig 2) and a table to the supplementary document S1 Table which shows from which hospitals the quantitative and qualitative data were taken, and the characteristics of these hospitals. References to these have been added in the text. |
| Although the use of more than one source of qual data (eg interviews and observation) increases robustness through triangulation it is not clear in the methods or the results how the observational data was used or triangulation was used. | Added to the methods: “*Our initial observations helped refine the topic guide*.” |
| It would also be useful to explain how the 3 observed hospitals were chosen eg) convenience vs particular characteristics to help understand the context of the data as this may change how this data should be considered in the overall picture. | Added to the methods when discussing the observations: “*to obtain an understanding of the paediatric admission process, and to inform the content of the topic guide; these hospitals were selected to observe settings with different levels of pulse oximetry adoption*”. |
| It would be good to increase the clarity or tighten the connection between the text description of the qualitative data and the labels used in the synthesis of the data in Figure 3 to make it easier to understand which parts of the text description relate to which part of this framework.  Some of this information is raised in the discussion eg) influence of protocols and guidelines and feedback from CIN data motivating improvement. If this came from the interviews - should it be part of the results rather than in the discussion (I may have missed it and apologise if I did)? | To tighten the connection between the text description of the qualitative data and the labels used in Figure 3, the following phrases from labels in Figure 3 (now called Fig 5) have been added to the text description of the qualitative data:  “*According to the interviews, HCWs are confident in their ability to use pulse oximeters*”  “*Guidance was also found to be influential*”  “*Other common environmental constraints include*”  “*The main individual level barrier to appropriate pulse oximeter use is due to skill constraints*”  The following phrases relating to old Figure 3 / new Fig 5 headings are already within the text description of the qualitative data: beliefs about pulse oximeters, intention to use pulse oximeters, use of a pulse oximeter, interpretation of pulse oximeter results  We moved the following text from the discussion to the results: “*HCWs frequently mentioned the Kenyan Basic Paediatric Protocols, appeared to trust them, and spoke about an increased awareness of the Protocols amongst the staff*.” |
| -Line 150 suggest "different health issues and treatments compared to older children"  -Line 234 onwards - suggest italics for quotes to make it clearer what is quote and text  -Line 363 In summary,... | Changes made as suggested |
| Line 44 Suggest rewording in findings: "...children with a reading below 90% were more than twice as likely to RECEIVE oxygen.... | This section has been changed to *“Children were more likely to be prescribed oxygen if a pulse oximeter reading was obtained... ”* |
| Line 263 "obtain oxygen" suggest "receive oxygen" | The phrase “*Children were more than twice as likely to obtain oxygen*” has been changed to “*The odds of a child being prescribed oxygen was more than twice as high*” |
| Line 216 For which children pulse oximeters are used - otherwise use a ? at the end of this theme heading. | Changed to “*Children with whom pulse oximeters are used*” |
| Line 225 I am not sure children "obtain" a pulse oximeter reading "have a pulse oximetry performed" "have a SpO2 recorded" | Changed to “*Pulse oximeters were also more likely to be used with children who*…” |
| Line 234 to 238 is a long and sometimes difficult to read sentence. Suggest breaking into more than one | We split the sentence into 3 sentences: “*According to the interview findings, many HCWs consider that pulse oximeters should be used with all children at admission. Others listed various “children who don’t need pulse oximeters”{8;Clinical Officer} and so for whom “doing an SpO2 doesn’t really make sense”{3;Medical Officer}. When these HCWs were asked which children do not need a pulse oximeter, the most commonly identified characteristic was those without respiratory symptoms, or more specifically, those with diarrhoea, vomiting, or physical trauma.*” |
| Line 254 "given a pulse oximeter reading" - the language around this needs to be tightened as per above. | Sentence changed to “*children with whom pulse oximeters were used had a 42% higher odds of being prescribed oxygen than children with whom pulse oximeters were not used*” |
| Line 265-6 I wonder if the sentence "suggesting HCWs are influenced by the recommended 90% threshold is more appropriate for discussion where it is already elaborated on. Alternatively this could be explicitly stated in Figure 3 in Influence of guidelines ie Oxygen for SpO2<90% (rather than referring generically to "guidelines") | Line 265 has been edited as discussed in response to one of your earlier comments. The guidelines referred to in Figure 3 is referring to a broader range of guidelines than just the 90% threshold, for example the Kenyan Basic Paediatric Protocols guidance on when to use a pulse oximeter |
| Line 273-274 suggest - follow clinical indications, either providing or withholding oxygen. | While we appreciate the attempt to make the language in this sentence more concise, we have kept the phrasing as is as we want to specify that these HCWs are relying on what the clinical signs (e.g. the outwardly presenting symptoms) seem to suggest and we think this is the clearest way to express this; also, given that the term ‘clinical indications’ is often used in relation to drugs, we want to refrain from using it in this context. |
| Please review sentence length throughout- sentences with a lot of brackets, colons and qualification sometimes lose their impact through their length | We have edited to reduce the length of sentences, and removed some of the brackets and colons. |
| Figure 3; In the box "self confidence in PD use: the second dot point needs revision for grammar | We have revised this bullet point. It now reads “*HCW considers they have the skills to use POs*” |
